# Development of a Custom Fluid Flow Chamber for Investigating the Effects of Shear Stress on Periodontal Ligament Cells

**DOI:** 10.3390/cells13211751

**Published:** 2024-10-23

**Authors:** Mustafa Nile, Matthias Folwaczny, Andreas Kessler, Andrea Wichelhaus, Mila Janjic Rankovic, Uwe Baumert

**Affiliations:** 1 Department of Orthodontics and Dentofacial Orthopedics, LMU University Hospital, LMU Munich, 80336 Munich, Germany; kfo.sekretariat@med.uni-muenchen.de (A.W.); mila.janjic@med.uni-muenchen.de (M.J.R.); 2 Department of Conservative Dentistry and Periodontology, LMU University Hospital, LMU Munich, 80336 Munich, Germany; matthias.folwaczny@med.uni-muenchen.de (M.F.); andreas.kessler@uniklinik-freiburg.de (A.K.); 3 Department of Prosthetic Dentistry, Faculty of Medicine, Center for Dental Medicine, Medical Center-University of Freiburg, University of Freiburg, 79106 Freiburg, Germany

**Keywords:** fluid flow, periodontal ligament, mechanical stimulation, cells, laminar, orthodontics, tooth movement, PDMS, tissue formation, shear stress

## Abstract

The periodontal ligament (PDL) is crucial for maintaining the integrity and functionality of tooth-supporting structures. Mechanical forces applied to the tooth during orthodontic tooth movement generate pore pressure gradients, leading to interstitial fluid movement within the PDL. The generated fluid shear stress (FSS) stimulates the remodeling of PDL and alveolar bone. Herein, we present the construction of a parallel fluid-flow apparatus to determine the effect of FSS on PDL cells. The chamber was designed and optimized using computer-aided and computational fluid dynamics software. The chamber was formed by PDMS using a negative molding technique. hPDLCs from two donors were seeded on microscopic slides and exposed to FSS of 6 dyn/cm^2^ for 1 h. The effect of FSS on gene and protein expression was determined using RT-qPCR and Western blot. FSS upregulated genes responsible for mechanosensing (FOS), tissue formation (*RUNX2*, *VEGFA*), and inflammation (*PTGS2/COX2*, *CXCL8/IL8*, IL6) in both donors, with donor 2 showing higher gene upregulation. Protein expression of PTGS2/COX2 was higher in donor 2 but not in donor 1. RUNX2 protein was not expressed in either donor after FSS. In summary, FSS is crucial in regulating gene expression linked to PDL remodeling and inflammation, with donor variability potentially affecting outcomes.

## 1. Introduction

Human tissues are inevitably exposed to ubiquitous extrinsic mechanical forces during everyday activities. Cells within different body tissues are sensitive to mechanical changes influencing their micro-environment and they react accordingly by adapting their cytoskeleton and the surrounding extracellular matrix [1]. These adaptational changes are central to maintain cellular physiology and function. Due to the key role of mechanical signals in tissue homeostasis, the topic of cellular mechanosensing, including molecular pathways involved in the mediation of mechanical signals, is receiving increasing attention [2].

In orthodontics, appliances are used to correct dental malocclusion and improve function. Such orthodontic appliances direct the modulation of the periodontium using controlled forces, thus facilitating tooth movement. The adaptational response of bone and periodontal ligament to orthodontic forces governs the movement of the tooth within the periodontium [3]. Such response has been described as bone formation at sites of tissue stretching and bone resorption at sites of tissue compression [3].

Apart from cells, i.e., fibroblasts, and different types of fibers, i.e., collagen, the interstitial fluid enclosed within the extracellular matrix comprises a major component of connective tissue, including the periodontal ligament. The interstitial fluid exchange between blood capillaries and lymphatics in tissues is maintained through the osmotic and hydrostatic pressure differences between these vessels and interstitial spaces. Changes in the geometrical shape of interstitial spaces in porous tissues have been suggested to influence the pattern of interstitial fluid movements and thereby expose the cells to non-physiological fluid flow shear stress (FSS) [2]. While orthodontic forces are perceived by the cell as stretching and compression, cells are also exposed to secondary shear stresses caused by interstitial fluid flow within PDL and bone, i.e., porous tissues [2]. Research has suggested that fluid movement is derived from changes in pore pressure gradient caused by matrix deformation [4,5]. While several studies have investigated the direct effect of compression and stretching on cells of the periodontium [6,7], only few studies have investigated the effect of FSS in this field [2]. Recent in vitro research compared the application of FSS of 1 and 6 dyn/cm^2^ at the cellular level [8]. However, correlating the magnitude of FSS to OTM is still a challenge: the thickness of the periodontal ligament (150–380 µm) requires specific proper techniques to measure the magnitude of FSS generated within the PDL in relation to the magnitude of applied forces exerted by the orthodontic appliances. As such the in vitro setup (parallel flow chamber) and the in vivo (i.e., clinical) situation (3D spatial arrangement, etc.) differ and are difficult to relate to each other.

Fluid flow direction and frequency within the biological tissues can vary from region to region, health and disease, and physiological movement [2]. The main fluid flow profiles were defined as steady laminar, pulsatile laminar, and oscillatory laminar [2,9]. Some studies have suggested that different fluid flow profiles may have a more cell-stimulating effect than others [9,10]. Moreover, cells might be able to discriminate between fluid flow profiles based on different physical activities like standing or walking [10,11].

Fluid flow and its changes can be detected by components of the cell membrane that connect the cell’s interior to the extracellular matrix. Different cell membrane mechanisms for transducing mechanical signals have been identified including, but not limited to integrins, connexins, glycocalyx, primary cilium, and ion channels [4,12,13]. As a result, various genes and metabolites are regulated including those responsible for tissue formation (e.g., *VEGFA*, *RUNX2*, *SP7*, and *TNFRSF11B*) and inflammation (e.g., *PTGS2/COX2*, *CXCL8/IL8*, and *IL6*) [2].

Different experimental fluid flow models have been established to study the effect of FSS in vitro. In a recent review, we identified studies that used in vitro setups such as parallel plate systems, rocking plate systems, orbital shaking systems, and plate and cone systems (cone viscometer) [2]. Based on the research question and the nature of mechanical fluid movement within the investigated tissue, the FSS setups were selected to provide the investigated fluid flow profile (steady, pulsatile, or oscillatory laminar). Each of these individual fluid flow setups is presented with advantages and/or limitations [2]. These can be attributed to the inherent constraints of the experimentally designed flow profile and the heterogeneity of mechanical stresses (precision) applied to cells, as exemplified by rocking plate and orbital shaking systems [14]. Nevertheless, parallel plate chambers connected to different driving devices, like peristaltic or syringe pumps, have been proposed to overcome such limitations and were customized according to specific objectives considered [2]. Unfortunately, commercially available parallel plate systems are commonly not only expensive but also require complex lab-side handling. Moreover, these systems primarily operate with a small sample volume, which impairs detailed functional downstream analysis of relevant pathways.

This study aimed to design and test a parallel plate fluid-flow apparatus to determine the effect of fluid shear stress on cells of the periodontium. Based on the currently available evidence on the character of fluid flow profile within bone and periodontal ligament during orthodontic treatment, one can assume that fluid flow within the periodontal ligament follows the direction of applied force [2]. This has been recently confirmed in silico for porous tissues such as bone and periodontal ligament [5]. The displacement of a tooth in the bony socket during the initial phase of orthodontic tooth movement (OTM) has been suggested to occur due to the compression/stretching of the periodontal ligament and the blood vessels [15,16]. The initial biphasic displacement has been proposed to occur within the first few hours [17,18]. These initial changes are followed by the longer lagging phase, which leads to the ultrastructural biological remodeling in the periodontium. It was the objective of this study (1) to design, construct, and test a parallel plate system to apply uniform FSS on human periodontal ligament cells (hPDLCs), specifically taking into consideration easy lab-side handling, affordable costs, and high sample volume for in-depth analysis of cell function. (2) To apply a physiologically relevant and standardized fluid flow profile and shear stress on hPDLCs to investigate the effect of FSS on OTM.

How this research will advance the field of knowledge: Though several studies highlighted the effect of FSS on tissue remodeling during OTM, this study should delineate the use of FSS as a potential remodeling element during OTM and develop an experimental method for detailed analysis of the effects of FSS on periodontal cells.

## 2. Materials and Methods

### 2.1. Design of the Parallel Flow Chamber

#### 2.1.1. Design Considerations

The principal design objectives were to construct a parallel flow chamber addressing the following characteristics:

1. Biocompatible material not affecting cellular functions, i.e., growth and viability.
2. Chamber material compatible with autoclave sterilization.
3. The chamber geometry allows for the loading and unloading of a standard microscopic slide (size: 76 × 26 × 1 mm^3^).
4. Easy assembly of the chamber not requiring special tools.
5. To ensure precise and robust performance, the design should ease seeding cells in a pre-defined area of uniform fluid flow.

**Figure 1:**
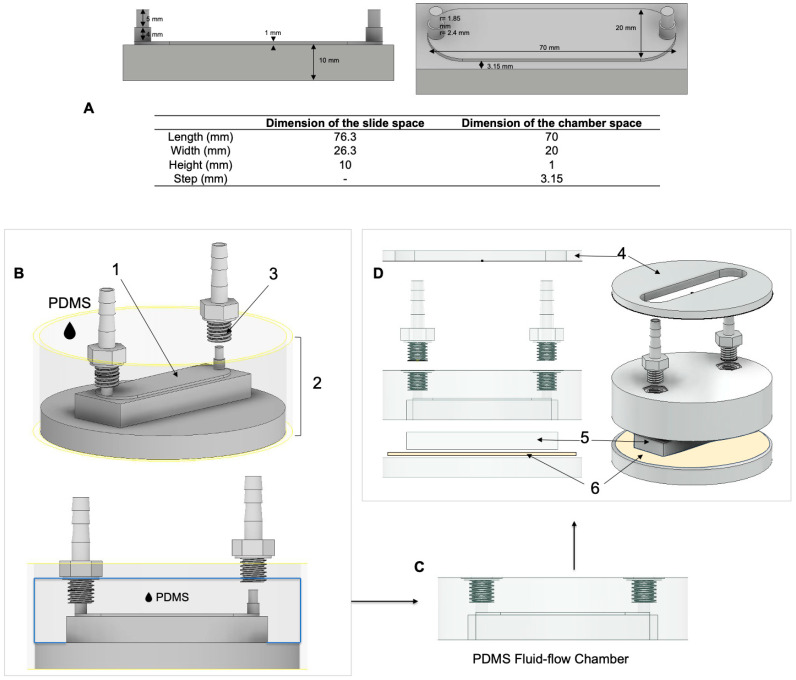
Chamber design and chamber production. (**A**) Inner chamber dimension. Dimensions of the master chamber model (identical to the inner part of the chamber). (**B**–**D**) Chamber production using the negative molding technique. (**B**) The master model of the inner chamber (1) was glued to the 3D-printed flask consisting of a base and frame (2) using modeling wax. Threaded nozzles (3) were placed onto the chamber’s inlet and outlet and degassed PDMS was poured into the flask. (**C**) The final chamber is made from PDMS with threaded fittings. (**D**) Exploded 3D image of the final chamber with all parts including chamber closing frame (4), chamber closing lid (5), and polyurethane nano tape (6). The parts not made from PDMS were 3D printed using an SLA printer.

To address the objectives related to chamber biocompatibility and sterilizability, polydimethylsiloxane (PDMS) was selected. The chamber itself was created using the negative molding technique. The fluid flow channel dimensions were 70 × 20 × 1 mm^3^ (length × width × height) (Figure 1A).

#### 2.1.2. Chamber Design, Computational Fluid Dynamic Simulations, and Construction

**Figure 2:**
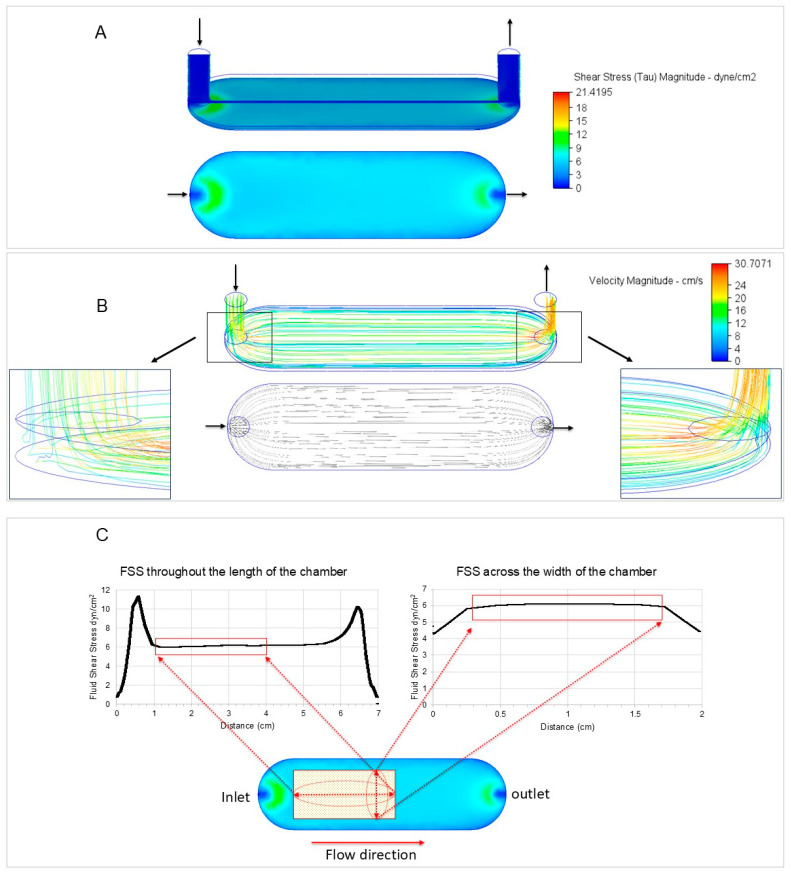
Computational fluid flow shear stress simulation of the parallel flow chamber using Autodesk CFD (Autodesk, San Rafael, CA, USA). (**A**) The shear stress magnitude was determined to confirm the mathematical calculations and distribution of wall shear stress at 6 dyn/cm^2^ across the desired chamber cell seeding area. Arrows represent the flow direction. (**B**) The velocity was simulated to determine undesirable phenomena such as turbulence. Streamline visualization of the flow field shows no flow turbulence at the seeding area of the flow chamber. Turbulence lines near the inlet and outlet are shown. (**C**) The region of consistent FSS was identified by fluid flow simulation (Autodesk CFD; Autodesk, San Rafael, CA, USA). The graphs depict FSS along the length (left) and across the middle of the chamber (right). A custom-made gasket was designed using Autodesk Inventor (Autodesk, San Rafael, CA, USA) (see Figure 3 for details).

The chamber was designed to ensure consistent, controlled fluid flow and minimal turbulence. Using a 3D modeling CAD software (Autodesk Inventor Professional 2022; Autodesk, San Rafael, CA, USA) and computational fluid dynamic (CFD) software (Autodesk CFG 2023; Autodesk, San Rafael, CA, USA), the channel dimensions and the fluid flow parameters were optimized, resulting in a setup that applies wall shear stress of 6 dyn/cm^2^ in a defined and reproducible manner. Herein, FSS magnitudes are reported in dyn/cm^2^, which can be converted to Pascal (Pa) using the following relationship: 10 dyn/cm^2^ = 1 Pa. Briefly, according to the size of a microscopic slide, a master model of the inner dimensions of the chamber (negative chamber model) was designed. The design was created to allow straightforward assembly to commercially available threaded connectors at the inlet and outlet as described below. CFD simulations were then done using the 3D chamber geometry (Figure 2A,B).

To manufacture the chamber, the CAD design was exported as an STL file and a master inner chamber template (inner part) was milled from polymethyl methacrylate (PMMA) guide material (InCoris; Dentsply-Sirona, Bensheim, Germany) with a dental 5-axis milling device (MCX5, minimum bur head diameter: 0.5 mm; Dentsply-Sirona, Bensheim, Germany). A custom-made molding flask that consisted of two components (base and frame) (Figure 1B) was designed and printed using SLA 3D resin printer (Form 3+; grey resin: RS-F2-GPGR-04; both Formlabs, Berlin, Germany). The molding flask components were assembled with the negative chamber model using melted modeling wax (pink modeling wax; DENTAURUM GmbH & Co. KG, Ispringen, Germany). Polydimethylsiloxane (PDMS) (SYLGARD™ 184 Silicone Elastomer Kit; Dow Corning, Midland, MI, USA) at a ratio of 1:5 was then thoroughly mixed and degassed in a vacuum chamber (Degussa Multivac^®^ compact, DeguDent GmbH, Hanau, Germany). To create the positive chamber model from PDMS, the degassed PDMS was then poured into the assembly, which consisted of the master inner chamber template glued to the 3D printed molding flask base, with polypropylene inlet/outlet nozzles positioned (G 1/8”-6 (1/4”) mm, polypropylene++, GT 186 PP; Landefeld Druckluft und Hydraulik GmbH, Kassel, Germany) (Figure 1B). The chamber closing lid was made by pouring PDMS at a ratio of 1:10 in a silicon chamber model (76.3 × 26.3 × 10 mm^3^) (part 5 in Figure 1D). The final assembly included all parts mentioned in Figure 1D in addition to a polyurethane nano tape (Foshan Opalus Adhesive Products Co., Ltd., Guangdong, China), which was used to provide an airtight seal (part 6 in Figure 1D). The chamber components were reciprocally secured with clamps.

The steady flow rate, *Q*, required to produce a constant fluid shear stress at the walls of a rectangular chamber (*τ*_wall_) is obtained according to:

$$\tau_{wall}=\frac{6\times \mu \times Q}{w\times h^{2}}\;=>\;Q=\frac{\tau_{wall}\times w\times h^{2}}{6\times \mu }$$

*w*, width (20 mm); *h*, height (1 mm); *τ*_wall_ = 6 dyn/cm^2^; *µ*, viscosity of medium (0.00072 Pa × s)

**Figure 3:**
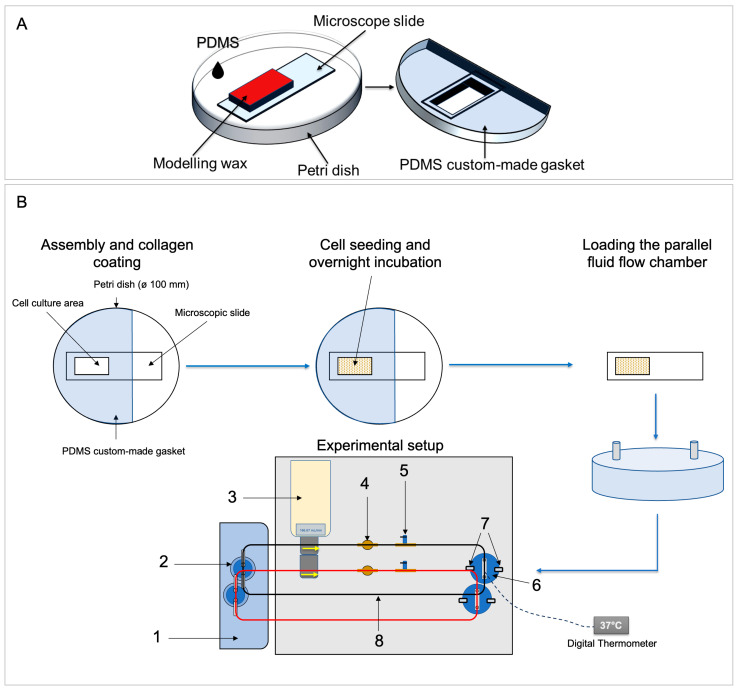
Workflow of the experimental setup. (**A**) Custom-made culture well gaskets. The gasket was constructed by blocking the desired area of the glass slide using modeling wax and molded with 1:10 PDMS in cell culture dishes. (**B**) First, the gaskets were placed onto microscopic slides and coated with collagen. Second, cells were seeded in the gasket well at a density of 3 × 10^5^ cells/cm^2^ and incubated overnight. Third, the slides were loaded into a parallel flow chamber and secured using clamps, after which the two chambers were stimulated in parallel. The complete setup consists of (1) a water bath used to keep the culturing medium temperature at ~37 °C; (2) culturing medium reservoir; (3) a peristaltic pump; (4) a pulse damper; (5) a bubble trap composed of a T-connector and a valve; (6) parallel flow chamber; (7) clamps; (8) silicon tubing (black: chamber 1; red: chamber 2).

A defined fluid viscosity of 0.00072 Pa × s (density: 1000 kg/m^3^) corresponding to cell culture medium with 10% serum was considered [19] and fluid shear stress of 6 dyn/cm^2^ was simulated using 166.67 mL/min fluid flow rate applying the parameters from the formula. The digital hall-effect flow sensor YF-S401 (model SEA, 5–24/V DC, Shenzhen SEA Technology Co., Ltd., Shenzhen, China) operated by a microcontroller (Arduino Uno) was used to confirm the output flow rate at the inlet during the pre-testing of the peristaltic pump. Graphs related to the velocity and distribution of wall fluid flow shear stress lengthways and obliquely within the chamber were generated (Figure 2). The regions of steady and constant fluid shear stress were identified from the graphs (Figure 2C). Based on this data, custom-made well gaskets for collagen coating and cell seeding were fabricated (Figure 3A).

#### 2.1.3. Custom-Made Gasket for Glass Slide Coating and Cell Seeding

The preferred area for collagen coating and cell seeding was selected based on the results of the computational simulation data (Figure 2C and Figure 3A). The final dimension of the cell seeding/coating area was 2.9 × 1.4 cm^2^ (~4.06 cm^2^). This area was then digitally designed using Autodesk Inventor and printed on a sheet of paper to be used as a template. Next, a glass microscopic slide was placed and superimposed on the printed template and the desired cell seeding/coating area was blocked using modeling wax of 7 mm thickness. Finally, the blocked glass slide was used as a negative model, i.e., it was placed in a petri dish and PDMS at a ratio of 1:10 was then poured into this petri dish to create the microscopic slide culture well gaskets (Figure 3A).

#### 2.1.4. Assembly of the FSS System and Temperature Adjustment

In addition to the flow chamber, the FSS system was completed with additional commercially available components (Figure 3B, Table 1). Test tube clips (0645.1; Carl Roth, Karlsruhe, Germany) were used to pinch the tubes during assembly and disassembly. The water heating bath (part 1 in Table 1) was used to keep the temperature of the circulating medium stable (details see below). The bubble trap (part 5 in Figure 3B) consisted of a barbed T-connector (part 5a in Table 1), and a stainless-steel lever air control valve (part 5b in Table 1).

**Figure 4:**
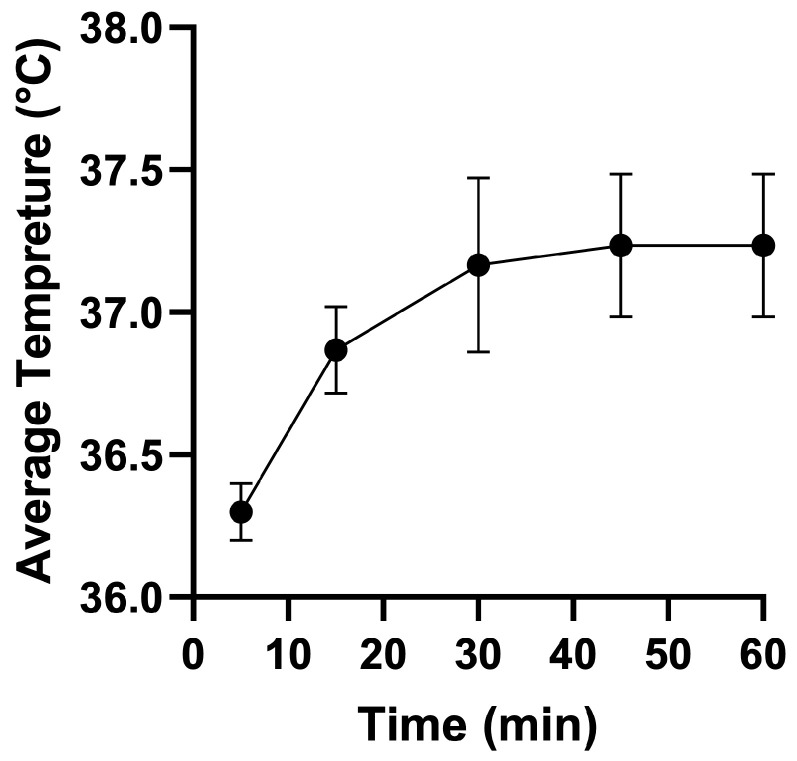
Calibration of the temperature in the parallel flow chamber. The temperature within the chamber was calibrated with the water heating bath using a digital thermometer implanted within the parallel flow chamber using a fluid flow rate of 166.67 mL/min.

The water heating bath (Table 1; Figure 3B, part 1) should keep the entire device at a constant temperature of ~37 °C throughout the tests. The temperature within the chamber was adjusted using a digital LCD thermometer (SPU:PYQ9143; ARCELI, China) calibrated with a standard mercury thermometer. The media reservoirs were prewarmed for 30 min in the water bath first. The probe of the electric thermometer was implanted into the chamber closing lid and then the dynamic flow temperature inside the chamber was measured. The measurements were repeated randomly between experiments to ensure a cell medium temperature between 36–37 °C at a fluid flow rate of 166.67 mL/min (Figure 4).

### 2.2. Cell Culture

Herein, human primary periodontal ligament cells (hPDLCs) and the human osteosarcoma cell line SaOS-2 (ACC 243; DSMZ, Heidelberg, Germany) were used. Initial testing of the FSS system and its performance was conducted with SaOS-2 cells. For the remaining experiments described, hPDLCs were used. These cells were obtained from the middle-third of premolar roots of two donors (donor 1: 14-year-old female, lower second premolars after orthodontic treatment; donor 2: 16-year-old male, lower first premolars before orthodontic treatment) extracted for orthodontic reasons according to established protocols [20,21]. The study was conducted in compliance with the Declaration of Helsinki. Approval for the collection and use of hPDLCs was obtained from the ethics committee of Ludwig-Maximilians-Universität München (project number 045-09). Informed consent was obtained from the patients and their legal custodians.

SaOS-2 cells and hPDLCs were both grown in Dulbecco’s Modified Eagle’s Medium/nutrient mixture F-12 Ham with HEPES (DMEM/F-12) (D6421; Merck/Sigma-Aldrich, Darmstadt, Germany). For cultivation of hPDLCs, this medium was supplemented with 10% FBS (FBS SUPERIOR stable, FBS.S 0615; Bio&SELL, Feucht/Nürnberg, Germany), 1% MEM vitamins (M6895; Merck/Sigma-Aldrich, Darmstadt, Germany), 1% GlutaMAX™ (100×; 35050061) and 1% antibiotic/antimycotic (100×, 15240062; both from Gibco Life Technologies, Carlsbad, CA, USA). For SaOS-2 cells, 15% FBS were used instead. Both cell types were grown in a humidified atmosphere with 5% CO_2_ at 37 °C and fed twice a week. Every 2 weeks, both cell types were passaged using 0.05% trypsin-EDTA solution (L 2143; Bio&SELL GmbH, Feucht/Nürnberg, Germany). For all experiments, hPDLCs of 3rd–5th passage and SaOS-2 of 6th–7th passage were used.

Lysates prepared from a growing HeLa cell culture were used as positive controls for Western blots. HeLa cells (300194; CLS Cell Lines Service GmbH, Eppelheim, Germany) were grown in T75 flasks using Dulbecco’s Modified Eagle’s Medium/nutrient mixture F-12 Ham with HEPES (D6421; Merck/Sigma-Aldrich, Darmstadt, Germany) supplemented with 10% FBS (FBS SUPERIOR stable, FBS.S 0615; Bio&SELL, Feucht/Nürnberg, Germany), 1% sodium pyruvate solution (S8636; Merck/Sigma-Aldrich, Darmstadt, Germany), 1% GlutaMAX™ (100×; 35050061) and 1% antibiotic/antimycotic (15240062; both from Gibco Life Technologies, Carlsbad, CA, USA). Cell lysates were prepared using RIPA buffer in the same way as described below (Section 2.7, sample preparation).

For fluid shear stress experiments, 500 mL of 1× DMEM FSS-medium was prepared from 10× DMEM (F 0455; Bio&SELL, Feucht/Nürnberg, Germany). The medium was supplemented with 10 mL HEPES (P05-01500; PAN-Biotech, Aidenbach, Germany), 5 mL GlutaMAX™ (100×), and 5 mL sodium pyruvate solution (S8636; Merck/Sigma-Aldrich, Darmstadt, Germany), and sterilized by filtering using a vacuum filter/storage bottle system (0.22 µm pore size; 431097; Corning, AZ, USA). The 1× DMEM FSS medium was then supplemented with 10% FBS and 1% antibiotic/antimycotic.

### 2.3. Coating and Cell Seeding

The custom-made microscopic slide culture well gaskets (Section 2.1.3 and Figure 3) were used to coat the defined area of sterile microscopic slides with rat tail collagen type 1 (50201; ibidi GmbH, Gräfelfing, Germany) according to the manufacturer’s “thin coating protocol”. Firstly, each sterile microscopic slide was mounted to the custom-made microscopic slide culture well gasket and placed in a petri dish. The collagen solution was diluted to 5 µg/cm^2^, pipetted in the well gaskets, and incubated for 1 h at room temperature under sterile conditions. Afterwards, the collagen solution was aspirated, and the coated area was washed twice with Dulbecco′s Phosphate Buffered Saline (DPBS) (D8537; Merck/Sigma-Aldrich, Darmstadt, Germany).

SaOS-2 cells or hPDLCs were expanded in T75 flasks. For FSS stimulation, the cells were resuspended in 1× DMEM FSS medium and then seeded at 3 × 10^5^ cell/cm^2^ on the collagen-coated slide region. To enhance cell attachment, incubation was continued overnight at 37 °C and 5% CO_2_ in a humidified atmosphere.

### 2.4. Preparation of a FSS Experiment

All components of the FSS system were decontaminated and disinfected as follows: (1) The medium in the media reservoirs was replaced with 70% ethanol and the FSS system was cleaned for 15 min using a flow rate of 50 mL/min. (2) Drain the system by opening the valve (bubble trap). (3) Flush with sterile double distilled water using a flow rate of 50 mL/min. (4) Drain the system. (5) Air dry overnight. (6) Wipe the outer surfaces using 70% ethanol. (7) Packaging each of the dissembled parts of the flow circuit in separate sterilization bags. (8) Autoclaving at 134 °C for 10 min. (9) The threaded polypropylene fittings were only disinfected by immersion in 70% ethanol, drained, and flushed again with sterile double distilled water.

### 2.5. Fluid Flow Shear Stress Application Using a Custom-Made Fluid Flow Apparatus

Once the closing frame was secured using clamps, the fluid flow apparatus was assembled (Figure 3B). The 1× DMEM FSS medium was driven by a peristaltic pump from a reservoir heated externally using a heating bath. The medium flowed through a pulse damper and bubble trap (with valve), into the chamber inlet, and exited through the outlet back to the reservoir.

For FSS application, hPDLCs were seeded on collagen-coated microscopic slides (see Section 2.3). After overnight incubation in the incubator (37 °C, 5% CO_2_, humidified atmosphere), the slides were removed from the gaskets and mounted into the custom-made parallel fluid flow chambers. Then, the chambers were carefully filled with 1× DMEM FSS medium. Assembly and disassembly of the fluid flow components were done under sterile conditions in a laminar flow cabinet.

Cells were subjected to steady laminar fluid flow shear stress of 6 dyn/cm^2^ (166.67 mL/min) for 1 h using a peristaltic pump with two pump heads (Shenchen Lab V3, Boading Shenchen Precision Pump Co., Ltd., Boading, China) (Figure 3B). The flow system was maintained at 36–37 °C using a water heating bath (Section 2.1.4). After 1 h of FSS, the slides were removed from the chambers and placed into slide dishes (quadriPERM^®^, 94.6077.307; Sarstedt, Nümbrecht, Germany) to prepare cell lysates (Section 2.7).

### 2.6. Cell Attachment and Cell Viability

Cell attachment was confirmed with light microscopic imaging before and immediately after FSS application. A total of five phase contrast images were captured using a 4× objective (EVOS^®^*fl*, Invitrogen, Carlsbad, CA, USA): one from each corner of the cell-seeded area and one from the middle.

Cell viability of hPDLCs after FSS application was assessed using the “Live/Dead Viability/Cytotoxicity Kit for Mammalian Cells” (L3224; Invitrogen, Carlsbad, CA, USA) according to the manufacturer instructions. Shortly, after 1 h FSS, the slides were transferred to a petri dish (628160, CELLSTAR^®^, Greiner Bio-One GmbH, Frickenhausen, Germany) and washed twice with DPBS (D8537; Merck/Sigma-Aldrich, Darmstadt, Germany). Then, the cell area was covered with a staining solution (2 µM calcein AM and 4 µM ethidium homodimer-1), wrapped with aluminum foil, and incubated for 30 min in the dark. Overall viability was checked and fluorescence micrographs of the center of the cell seeding area were taken using a fluorescence microscope (EVOS^®^*fl*, Invitrogen, Carlsbad, CA, USA) with a 10× objective.

### 2.7. Sample Preparation

Following 1 h FSS, the cell culture supernatants were removed, and the cells were washed twice with sterile PBS. Subsequently, cell lysates were prepared for either (1) total RNA preparation or (2) Western blot detection, as follows. For total RNA isolation, cell lysates were prepared from each slide using 1000 µL of RNA lysis buffer (R0160-1-50; Zymo, Irvine, CA, USA) supplemented with 40 µL DTT (1 M DTT solution; A3668,0050, PanReac AppliChem, Darmstadt, Germany) as previously described [20,21]. Until sample collection was completed, these cell lysates were stored at −80 °C. For Western blot detection, PDL cells were lysed using ice-cold RIPA buffer (Pierce™ RIPA lysis buffer, 8900; Thermo Fisher Scientific, Waltham, MA) supplemented with 1× Halt™ Protease Inhibitor cocktail (1862209; Thermo Fisher Scientific, IL, USA) and incubated for 30 min on ice. The RIPA lysates were centrifuged at 14,000× *g* at 4 °C for 5 min to obtain cell extracts and stored at −80 °C until analysis.

### 2.8. Quantitative Reverse-Transcriptase Polymerase Chain Reaction (RT-qPCR)

#### 2.8.1. Primer Selection

Gene expression of *PTGS2/COX2*, *IL6*, *FOS*, *RUNX2*, *CXCL8/IL8*, and *VEGFA* after exposure of cells to FSS was determined using RT-qPCR. A checklist adhering to the “Minimum Information for Publication of Quantitative Real-Time PCR Experiment” (MIQE) [22,23] standards is provided in Supplement File S1.

Primer sequences for both target and potential reference genes were sourced from previous publications (Table 2, Supplement File S1). All primer pairs used in this study underwent in silico testing according to the MIQE guidelines [22]. Unmodified primers were synthesized by commercial sources (TIB Molbiol Syntheselabor GmbH, Berlin, Germany; Metabion GmbH, Planegg/Steinkirchen, Germany; both: OPC^®^ purification). The optimal annealing temperatures were ascertained through gradient PCR, utilizing the qPCR cycling conditions outlined in the MIQE checklist (Supplement File S1). To verify primer specificity, agarose gel electrophoresis was conducted. The efficiency of the primers was assessed by constructing standard curves from cDNA serial dilutions and quantified using the LightCycler^®^ 480 with the primer pairs specified. In addition to the previously published primers [20,21], the specificity and sensitivity of the *CXCL8*/*IL8* and *VEGFA* primers were validated by gradient PCR and qPCR. To evaluate the primer efficiencies, standard curves prepared from serial dilutions of cDNA (undiluted, 1:10, 1:100, 1:1000, and 1:10,000 or undiluted, 1:2, 1:4, 1:8, 1:16) were quantified with the LightCycler^®^ 480 using the primer pairs as listed (Table 2, Supplement File S1).

#### 2.8.2. Reference Genes

Reference gene sReference Geneselection was based on a set of previously published reference genes (*EEF1A1*, *GAPDH*, *POLR2A*, *PPIB*, *RNA18SN5*, *RPL0*, *RPL22*, and *YWHAZ*) [21] (Supplement File S1). For each potential reference gene, RT-qPCR was conducted using cDNA samples subjected to 1 h FSS and corresponding control samples. The initial Cq values (Supplement File S2) were analyzed with RefFinder [24,25], and the most consistent genes were identified as reference genes for RT-qPCR [22].

#### 2.8.3. RT-qPCR Procedure

After 1 h FSS, the fluid flow chambers were dissembled and the slides were placed in a slide dish (quadriPERM^®^, 94.6077.307; Sarstedt, Nümbrecht, Germany). Cell lysates were prepared as described in Section 2.7 on sample preparation.

For cDNA synthesis, 600 ng total RNA was reverse transcribed in a total volume of 20 µL (SuperScript™ IV First-Strand Synthesis System, 18091050; Thermo Fisher Scientific, Waltham, MA, USA). Subsequent quantitative PCR (qPCR) was done as described previously [20,21] with the following modifications: the cDNA was pre-diluted 1:10 with sterile, ultrapure water and 5 µL of the diluted cDNA was amplified in a total volume of 20 µL. Quantitative PCR was performed according to the manufacturer’s instructions on a LightCycler^®^ 480 system equipped with LCS480 software version 1.5.0.39 (Roche Molecular Diagnostics, Basel, Switzerland). Each qPCR reaction contained 5 μL of the pre-diluted cDNA, 2 μM of each specific forward and reverse primer, and 10 µL of the Roche LightCycler^®^ 480 SYBR Green I Master (04887352001; Roche Diagnostics GmbH, Mannheim, Germany). Subsequent melting curve analysis was conducted post-PCR to monitor the specificity of the PCR amplification. After averaging (geometric mean) the reference genes (*RPL0*, *RPL22*) for a given donor/condition combination, the 2^−ΔΔCq^ method was applied for the quantification of gene expression [26,27]. Each of the four biological replicates of a donor (FSS and corresponding control sample), was analyzed twice with RT-qPCR. The technical replicates of each donor/condition combination were averaged and used for statistical analysis. Data were presented as mean fold change.

### 2.9. Western Blotting

For Western blot analysis, the RIPA cell lysates were defrosted, and the total protein concentration was determined using the Pierce^TM^ BCA Protein Assay Kit (23227; ThermoFisher Scientific, IL, USA). From each lysate, 10 µg total protein was separated using 14% precasted gels (43269.01, SERVAGel^TM^ TG PRiME 14%; SERVA Electrophoresis GmbH, Heidelberg, Germany). In parallel, for each protein of interest, positive controls were separated on the gels: human cyclooxygenase-2/COX-2 baculovirus-insect cells overexpression lysate (12036-H08BL; Sino Biological Europe GmbH, Eschborn, Germany) and HeLa cell lysate as a positive control for GAPDH. The Western transfer was done onto 0.2 µm PVDF membranes (ISEQ00010, Immobilon^®^-P^SQ^ Transfer Membrane; Merck Millipore Ltd., Country Cork, Ireland). The PVDF membranes were blocked with 3% bovine serum albumin (8076.2 Albumin Fraction V; Carl Roth GmbH, Karlsruhe, Germany) in TBS-T buffer for 1 h at room temperature. Subsequently, the membranes were incubated overnight at 4 °C with the following specific primary antibodies (all antibodies were from R&D Systems/Bio-Techne, Minneapolis, MN, USA): monoclonal mouse anti-human/mouse/rat GAPDH antibody (1:10,000; MAB5718), monoclonal mouse anti-human COX-2 antibody (1:500; MAB4198), and polyclonal rabbit anti-human/mouse/chicken/equine/primate RUNX-2 antibody (1:800; NBP2-24755). After washing the PVDF membranes three times with TBS-T, they were incubated with either of the horseradish peroxidase (HRP)-conjugated secondary antibodies (HAF007, 1:1000, goat anti-mouse IgG HRP-conjugated antibody; HAF008, 1:1000, anti-rabbit IgG HRP-conjugated antibody). After each detection cycle, the membranes were washed trice with TBS-T for 5 min and then reprobed with a different primary/secondary-antibody combination. Immunoreactive bands were visualized using an enhanced chemiluminescent (ECL) system (34078, SuperSignal^®^West Pico Chemiluminescent Substrate, Thermo Fisher Scientific, IL, USA). Chemiluminescent detection was done using Chemi-Smart 5000 (PeQLab, Biotechnologie GmbH, Erlangen, Germany).

### 2.10. Statistics

Descriptive statistics of the gene expression results are reported as mean ± standard deviation (SD), median, and minimum/maximum for each of the two donors separately. For each gene locus, the data of four biological replicates, each representing the average of its two technical replicates for each experimental condition/donor combination, were included in the analysis. For each gene locus, differences between the FSS application and the corresponding control were evaluated using the Mann-Whitney U-test. All statistical procedures were performed using IBM SPSS Statistics 29 (IBM Corp., Armonk, NY, USA). All test procedures were two-tailed considering *p* < 0.05 significant. The figures were prepared with GraphPad Prism (version: 8.0.1; GraphPad Software, La Jolla, CA, USA).

## 3. Results

### 3.1. Cell Attachment

**Figure 5:**
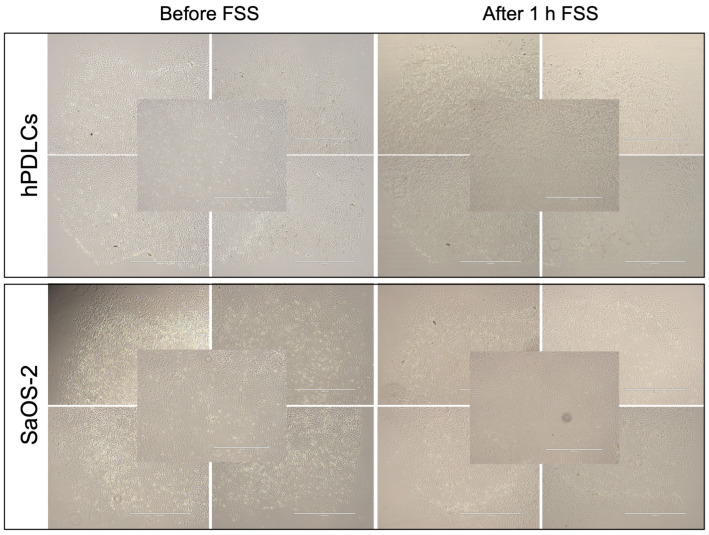
Cell attachment of human periodontal ligament cells (hPDLCs) and human osteosarcoma cell line (SaOS-2) was assessed by microscopy before and after applying FSS. Microscopic images of cells growing in the corners and center of the seeding area of the microscopic slide are shown. (Scale bar: 1000 μm).

To determine cell attachment after 1 h of FSS, the experimentally employed slides were microscopically inspected before and after FSS application. Overall, minimal detachment of hPDLCs and SaOS-2 cells from the collagen-coated microscopic slide area was observed. However, the cell attachment of SaOS-2 cells was better than that of hPDLCs (Figure 5).

### 3.2. Cell Viability

**Figure 6:**
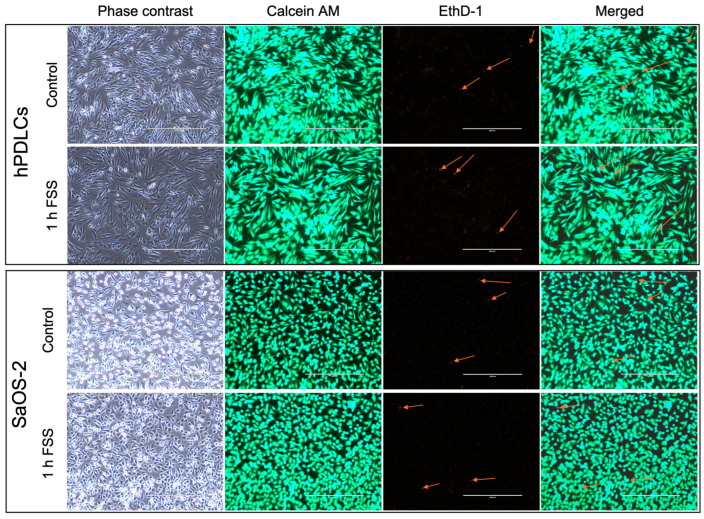
Cell viability of human periodontal ligament cells (hPDLCs) and human osteosarcoma cell line (SaOS-2) was assessed by live/dead cell staining. Microscopic images of cells growing in the center of the seeding area of the microscopic slide are shown. Live cells are indicated by calcein AM staining (green), and dead cells are indicated by ethidium homodimer-1 (EthD-1) staining (red arrows). (Scale bar: 400 μm).

A live/dead assay was used to determine cell viability after 1 h FSS (Figure 6). Overall, the results show that the viability of hPDLCs and SaOS-2 cells remained largely unaffected, with only a small portion of non-vital cells following exposure to mechanical stress.

### 3.3. Reference Gene Selection

**Figure 7:**
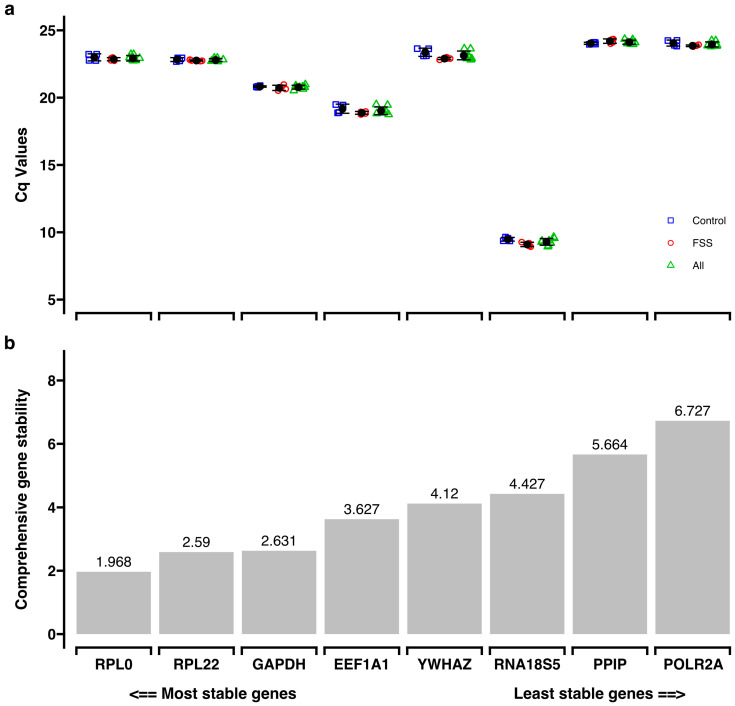
Reference gene primer stability was assessed using RefFinder [25]. (**a**) Descriptive statistics of the Cq values of the reference gene panel (FSS: 1 h FSS) (n = 4); Control: negative control (n = 4); All: FSS and control groups combined (n = 8). (**b**) The result from the comprehensive analysis of gene stability for the reference gene panel from RefFinder. Lower values in this analysis correspond to higher gene stability.

To validate reference genes, the expression of eight pre-selected genes was determined using RT-qPCR on samples subjected to FSS for 1 h and their corresponding controls. *RNA18S5* proved to be the most abundant reference gene, with a mean Cq value of 9.29 ± 0.248 (Figure 7; Supplement File S2). To identify the most stable reference genes within the panel, comprehensive gene stability values based on four different algorithms were calculated using the RefFinder program [25] (Supplement File S2). Consequently, *RPL0* (gene stability: 1.968), and *RPL22* (gene stability: 2.59) were selected as the most stable reference genes tested (Figure 7), in accordance with the MIQE guidelines [22].

### 3.4. Target Gene Expression

Fluid shear stress was applied to hPDLCs at 6 dyn/cm^2^ for one hour. Subsequently, the expression levels of target genes were assessed using RT-qPCR on the transcriptional level, with *RPL0* and *RPL22* serving as reference genes. The study encompassed six specific genetic loci, which represented three distinct functional categories: genes related to tissue remodeling (i.e., *RUNX2*, *VEGFA*), the mechanosensing-associated gene *FOS*, and inflammation-related genes including *PTGS2*/*COX2*, *CXCL8*/*IL8*, and *IL6*. Relative gene expression (mean fold changes, FC) between test groups and their respective controls was determined (Table 3).

#### 3.4.1. FSS Upregulate the Mechanosensitive *FOS* Gene

**Figure 8:**
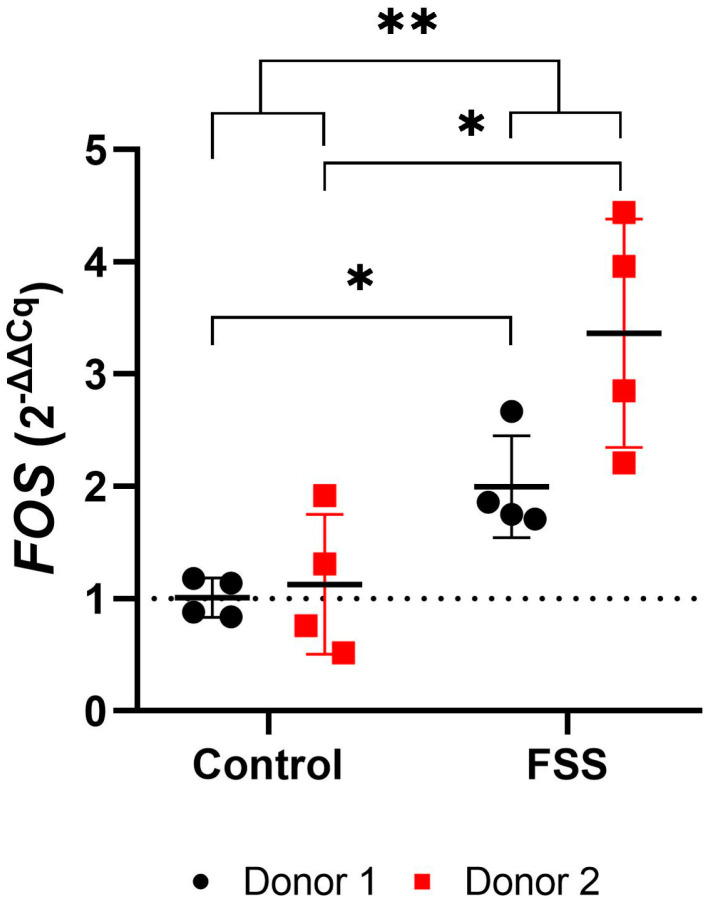
Gene expression of the early mechanosensitive responder *FOS* after 1 h fluid shear stress. Each test group is represented by the mean (━), with error bars that indicate the standard deviation (SD). The 2^−ΔΔCq^ technique was used, with *RPL0* and *RPL22* as reference genes. The differences between the test and control groups were evaluated using the Mann-Whitney U Test. Groups with significant differences are highlighted as follows: * *p* < 0.05; ** *p* < 0.01.

*FOS* gene expression significantly increased in hPDLCs for both donors compared to controls after one-hour exposure to FSS (mean total FC: 2.68; Figure 8, Table 3). Upregulation of genes was stronger in hPDLCs of donor 2 as compared to donor 1 (mean FC; donor 1: 2.00; donor 2: 3.37) (Figure 8, Table 3).

#### 3.4.2. FSS Upregulate Genes Responsible for Inflammation

**Figure 9:**
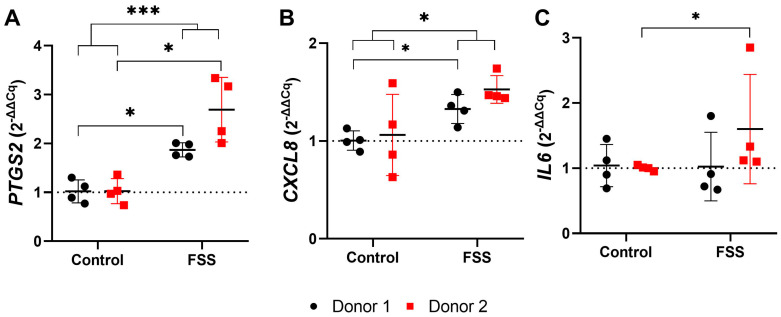
Gene expression of inflammation-related genes after 1 h fluid shear stress: (**A**) *PTGS2*, (**B**) *CXCL8* (*IL8)*, and (**C**) *IL6*. Each test group is represented by the mean (━), with error bars that indicate the standard deviation (SD). The 2^−ΔΔCq^ technique was used, with *RPL0* and *RPL22* as reference genes. The differences between the test and control groups were evaluated using the Mann-Whitney U Test. Groups with significant differences are highlighted as follows: * *p* < 0.05; *** *p* < 0.001.

One-hour FSS significantly increased *PTGS2* and *CXCL8/IL8* gene expression in cells of both donors compared to control (mean total FC; *PTGS2*: 2.28; *CXCL8*: 1.43) (Figure 9, Table 3). Moreover, a high degree of inter-donor variability was found for human periodontal ligament cells regarding the expression of *PTGS2* (mean FC; donor 1: 1.87; donor 2: 2.69) and *CXCL8* (mean FC; donor 1: 1.33; donor 2: 1.53) (Figure 9, Table 3). Similarly, one-hour 6 dyn/cm^2^ FSS induced stronger *IL6* gene expression in hPDLCs of donor 2 than donor 1 (mean FC; donor 1: 1.03; donor 2: 1.60) (Figure 9, Table 3).

#### 3.4.3. FSS Upregulate Genes Responsible for Tissue Formation

**Figure 10:**
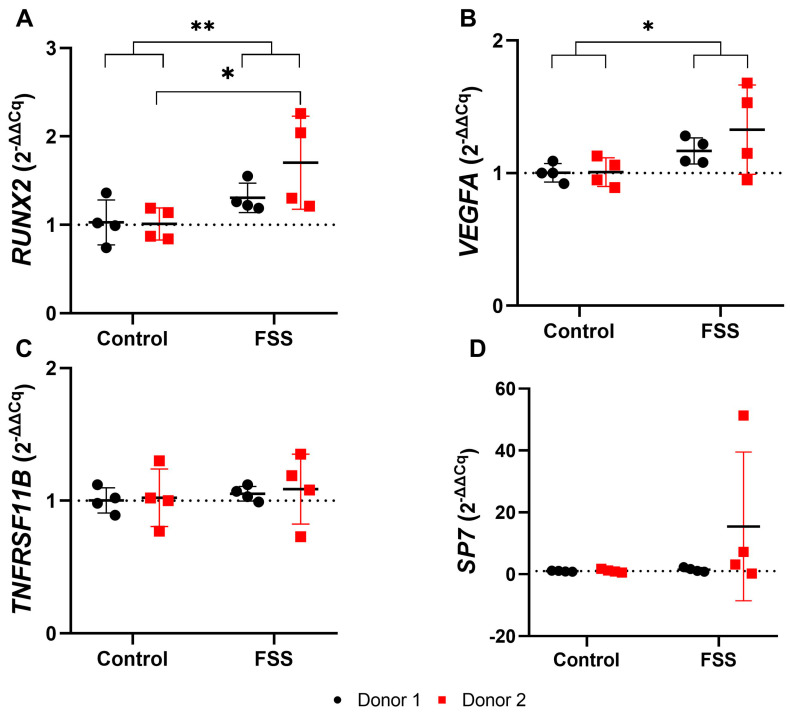
Gene expression of osteogenic differentiation-related genes after 1 h fluid shear stress: (**A**) *RUNX2*, (**B**) *VEGFA*, (**C**) *TNFRSF11B*, and (**D**) *SP7*. Each test group is represented by the mean (━), with error bars that indicate the standard deviation (SD). The 2^−ΔΔCq^ technique was used, with *RPL0* and *RPL22* as reference genes. The differences between the test and control groups were evaluated using the Mann-Whitney U Test. Groups with significant differences are highlighted as follows: * *p* < 0.05; ** *p* < 0.01.

Exposure to 6 dyn/cm^2^ FSS for one hour led to a generally significant increase in the expression of *RUNX2* and *VEGFA* (mean total FC; *RUNX2*: 1.50; *VEGFA*: 1.25) in hPDLCs of both donors (Figure 10, Table 3). Again, hPDLCs from donor 2 showed a significantly stronger amplification of *RUNX2* than those from donor 1 (mean FC; donor 1: 1.31; donor 2: 1.70), while no significant difference was shown between donors for *VEGFA* (mean FC; donor 1: 1.17; donor 2: 1.33) (Figure 10, Table 3).

#### 3.4.4. Western Blot Analysis

**Figure 11:**
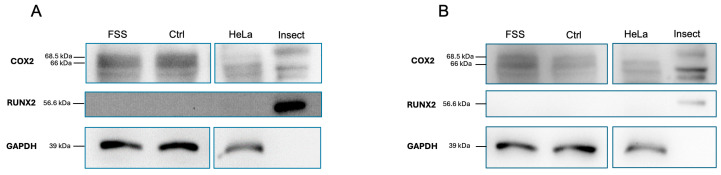
Western blot analysis of PTGS2/COX2 and RUNX2 proteins after 1 h fluid shear stress. FSS induced the expression of PTGS2 but not RUNX2. Lysates from donor 1 (**A**) and donor 2 (**B**) from 1 h FSS, the corresponding control (Ctrl), and the positive controls for GAPDH (HeLa), COX2, and RUNX2 (both expressed in baculovirus-insect cells) were separated by PAGE on a 14% SDS gel and transferred onto a PVDF membrane.

The obvious transcriptional effects of 1 h 6 dyn/cm^2^ FSS did not, however, result in translational changes of RUNX2 in hPDLCs (Figure 11). However, there was an increase in PTGS2/COX2 protein expression compared to the control. The molecular weights of RUNX2 (56.6 kDa), PTGS2/COX2 (68.5 and 66 kDa), and GAPDH (39 kDa) were determined in relation to the molecular weight marker and confirmed with the positive controls (HeLa cells for GAPDH and baculovirus-insect cells for RUNX2 and PTGS2/COX2). Full details concerning the Western blot experiment can be found in Supplement File S3.

## 4. Discussion

The construction of the parallel flow chamber described herein, aimed to address simplicity and cost-effectiveness, and to expose cells to steady laminar fluid flow shear stress to mimic rheological phenomena as proposed within the periodontal ligament. The design proved ease of assembly/disassembly and sterilizability, absence of medium loss due to leakage through tubing or evaporation, and homogeneous distribution of shear stress magnitude along the selected area through CFD analysis. In addition to the proposed main design goals that have been achieved, the objectives as stated previously in terms of establishing a robust in vitro setup to determine the effects of FSS caused by OTM are discussed as follows.

### 4.1. Selection of FSS Parameters and Justification of the Experimental Setting

Orthodontic treatment is centrally based on applying well-defined therapeutic forces to the tooth to induce movement and correct malposition. Interstitial fluid flow occurs due to a change in PDL pore pressure caused by the displacement of the tooth within its alveolar socket [2]. Previous in silico studies estimated a FSS range between 8–30 dyn/cm^2^ within bone lacunar canaliculi [28]. However, it is suggested that the physiological FSS occurring within the periodontal ligament during everyday loads is smaller than in bone, due to its larger porosities and higher resilience than bone [13]. By utilizing finite element models of collagen fibrils found in ligaments and tendons, earlier research estimated the magnitude of FSS between 0.2–12.1 dyn/cm^2^ [29]. This approximation of FSS is linked to alterations in geometrical pore size, which is determined by the ratio of interfibrillar space to the overall extracellular matrix volume. Current in vitro studies have suggested that 6 dyn/cm^2^ FSS of short duration is associated with periodontal ligament cell proliferation [8]. Also, it was concluded that fluid flow shear stress is associated with tissue formation [2].

**Figure 12:**
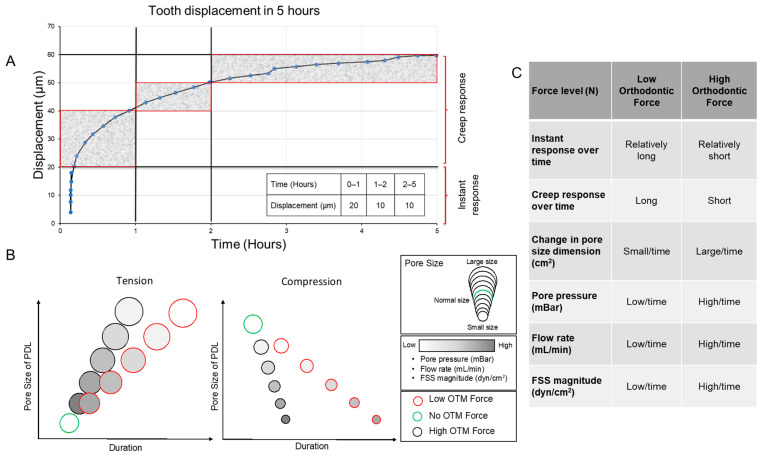
Force-related tooth displacement alters PDL dynamics (pore size, pore pressure, flow rate, and FSS). (**A**) The amount of displacement was presented in a table. As illustrated, the greatest displacement happens during the first hour using a force of 1 N [modified from [18]. (**B**,**C**) Conceptual representation of FSS magnitude during low and high orthodontic force. By applying low orthodontic force, the duration of fluid flow will be longer with a lower FSS magnitude over time and vice versa.

Assessing the fluid flow duration during orthodontic force application is challenging. Yet, the optimum force range during OTM has been suggested from 0.3 to 1 N [30,31]. Based on the observations from in vivo and in silico experiments on force-related tooth displacement [5,18], two phases, the early “instant displacement” and the later “creep displacement” (hydro-mechanical coupling effect) have been defined (Figure 12A). The initial phase lasts for <4 s and corresponds to the mechanically induced unfolding of the collagen fibers [18,32]. The later second phase continues for roughly 5 h and is related to the damping effect of the interstitial fluid, acting as a hydraulic damper during mechanical tooth loading [17,18,33].

Herein, cells were subjected to FSS for one hour. This choice was informed by two main factors: first, the application of 1 N force to a tooth leads to the highest level of force-induced tooth displacement within the first hour [18]. This displacement is primarily attributed to changes in fluid content (“creep”) within the periodontal ligament. Second, this specific timeframe has been extensively explored in various experimental setups [2].

While orthodontic forces induce therapeutic tooth movement through displacement of the tooth within the alveolar socket, it is also important to consider the transient effects of bite force on tooth displacement. Recent research has suggested that more intensive bite forces are associated with a stronger tooth displacement within the socket over time [34]. The same study indicated that a longer application of bite force is associated with a longer cumulative tooth displacement. Consequently, it is anticipated that a lower force magnitude would be associated with a longer tooth displacement duration and, therefore, lower FSS (Figure 12B). In addition, we suggest that while the displacement of the tooth is associated with continuous change in the pore size, pore pressure, flow rate, and therefore FSS (Figure 12B,C), it is difficult to have a comprehensive setup that discloses the dynamic changes of all these parameters over time in a controlled setup.

### 4.2. Chamber Construction

To determine the effect of FSS on cells, different experimental approaches have been applied, such as plate shakers, orbital shakers, cone and plate systems, or motorized custom-made flow chambers [2].

Parallel-plate flow chambers are commonly used to determine the effect of fluid flow shear stress on cells [2]. Different channel designs, including substrate topography [35] or cross-section geometry have been implemented to mimic specific flow conditions [36,37]. Custom-made fluid flow systems are mostly used to determine the effect of fluid shear stress on bone, periodontal ligament, and mesenchymal stem cells [2].

Herein, a custom-made parallel-flow chamber that can be loaded with a microscopic slide was designed and connected to a custom-made setup to determine the effect of steady laminar fluid flow on cells of the periodontium. The main goal was to construct a chamber that could accurately stimulate cells and yield enough of the sample for downstream analysis. The construction of a custom-made well gasket was based on a prior computational simulation identifying the region of constant and reproducible shear stress for optimum cell seeding. Addressing biocompatibility, ease of assembly/disassembly, and the possibility of multiple uses, PDMS and polypropylene have been used due to their high compatibility with cell growth and function [38,39].

The inherent risk of leakage has been described as one of the major drawbacks of previously suggested fluid flow systems [2]. We eliminated this issue by implementing threaded connectors at the inlet and outlet and maintaining minimal inlet pressure by designing a flat setup, where the reservoir is at the same level as the chamber to minimize the build-up of the inlet pressure [40]. Uncontrolled fluid pressure might result in cell deformation and deflection of the culturing substrate [40]. Higher pressure levels within the chamber lead to deflection of the microscopic slide ultimately causing additional uncontrolled mechanical stimuli [40].

Herein, a peristaltic pump was used to create fluid flow with a sufficient volume of cell culturing media for at least one hour. A standardized laminar flow was maintained by an additional pulse damper. In several previous reports, syringe pumps have been used instead [2]. However, the considerably larger chamber volume and the intended fluid flow rate would have required at least two syringe pumps with specific valves and tubing connections to create unidirectional laminar FSS. Moreover, a larger chamber would require a higher flow rate to achieve the desired stress level, which would affect the syringe’s medium-volume capacity. Therefore, a peristaltic pump was selected for our design in addition to a pulse damper to ensure sufficient circulating cell culturing media during the entire duration of the experiment of one hour while maintaining steady laminar flow.

The constructed chamber herein can sustain long-term continuous use, once it is securely assembled. This has been verified during several pre-tests done for temperature calibration lasting for up to six hours without leakage. The longest tested FSS-duration lasted for 2 h with SaOS-2 cells. Microscopic inspection revealed that the cells remained attached to the glass slide with optimal confluency.

### 4.3. Temperature

Maintaining appropriate temperature conditions using a flow chamber without an incubating device has already been described [41,42]. A water heating bath has been used to keep the culture medium within the flow chamber constant. Due to residual fluid within the flow tubing and pulse damper, the water bath temperature was calibrated according to the temperature of the medium that was entering the parallel flow chamber.

Before the cell culture experiments, the temperature was measured for 1 h using a thermometer implanted within the chamber room. At a fluid flow rate of 166.67 mL/min, the temperature within the fluid flow chamber gradually increased from 36.3 to 37 °C within the first 20 min, reaching a steady state for the remaining 40 min at ~37 °C.

### 4.4. Cell Viability and Attachment

The periodontal ligament (PDL) is situated between the tooth and alveolar bone and contains various types of cells within a matrix primarily composed of collagen types 1 and 2 [43,44]. Cells can recognize and bind to collagen through specific receptors, particularly integrins, forming focal adhesions. Collagen coating has been used frequently in biomedical applications to enhance surface biocompatibility by mimicking the natural extracellular matrix (ECM) [45,46]. Research has found that collagen coating improves surface biocompatibility of PDMS microfluidic chambers by increasing the hydrophilicity of the surface, thus improving cell adhesion, proliferation, and uniformity of mesenchymal stem cells [47]. Similarly, collagen coating of dental implants has shown improved surface biocompatibility through better fibroblast cell attachment [48]. Various methods, such as centrifugation and flow chambers, have been introduced to measure the strength of cell adhesion to biomaterials and are suggested as indicators of the biocompatibility of different coatings [49].

Overall, cell attachment and viability remained unaffected after exposure to 1 h of FSS. Nevertheless, cell attachment was not significantly affected in either cell type, indicating that these cells were able to adapt to and withstand mechanical forces and were resilient under the controlled dynamic conditions applied.

### 4.5. Expression of Target Genes

The periodontal ligament (PDL) is a connective tissue that plays a crucial role in tooth support and is sensitive to mechanical stimuli. The hPDLCs are exposed to mechanical forces, including FSS, due to occlusal loads induced by masticatory activity or OTM [17]. Fluid shear stress refers to the frictional stress exerted by fluid flow on the surfaces of cells. The loss of mechanical stimuli has been linked to the atrophy of the PDL and substantial resorption of alveolar bone [50]. Conversely, an excessive mechanical load results in the resorption of alveolar bone support [51]. Herein, the effect of 6 dyn/cm^2^ FSS on hPDLCs has been determined in vitro using the flow chamber as described above. As hPDLCs have been shown to behave similarly to mesenchymal stem cells in terms of force-related osteogenic differentiation [52,53], we propose that FSS could regulate hPDLCs signaling towards osteogenic differentiation.

#### 4.5.1. Effect of FSS on Mechanosensing

In the current study, FSS upregulated *FOS* gene expression in hPDLCs of both donors, despite showing considerable interindividual differences. The *FOS* gene encodes a subunit of the dimeric transcription factor AP-1 (activator protein 1) family, which is involved in the regulation of proliferation and differentiation of osteoblasts [54]. Several genes associated with osteoblastic differentiation and the synthesis of extracellular matrix are under the control of AP-1, i.e., RUNX2, collagenase-3, or osteocalcin [55,56]. Also, FOS has been found to regulate the FSS-associated expression of the *PTGS2* gene, which is centrally involved in the adaptive response of bone to mechanical stimuli [57]. An increased expression of Fos and FosB RNA resulting from mechanical loading of osseous tissue in animals was found to enhance bone formation [58,59]. Based on the current results, the FSS-induced upregulation of *FOS* gene expression seems to be involved in OTM-associated bone remodeling.

#### 4.5.2. Effect of FSS on Osteogenic Differentiation

FSS also induced an upregulation of *RUNX2* and *VEGFA*, with considerable differences between both donors. On the contrary, FSS did not cause changes in the expression of *SP7* and *TNFRSF11B/OPG*.

The Runt-related transcription factor 2 (*RUNX2*) is considered one of the key regulators of the differentiation of mesenchymal stem cells into osteoblasts. Previous data have shown that FSS upregulates the *RUNX2* gene in hPDLCs leading to osteoblastic differentiation [52]. This process has been suggested to occur through the activation of the *ERK1/2* and *MAPK* pathways [60,61]. The differentiation of pre-osteoblasts to fully functioning osteoblasts requires the zinc finger-containing transcription factor 7 (*SP7*) [62]. While no changes in *SP7* expression after 1 h exposure to FSS were found herein, significant upregulation of *SP7* was previously reported after a period of >6 h following FSS exposure [52]. On the protein level, the increased gene expression of *RUNX2* was not reflected by an increased RUNX2 protein level. Most likely this might be explained by the delay between increased transcription and the later resulting translation into protein, which might occur not before periods of 1 h. In addition, the observed discrepancy between transcriptional upregulation and protein expression can be attributed to the time between FSS stop and cell lysate preparation, which was ~60 s. Further research may consider a pulse-chase experimental setting while minimizing the required time for cell lysate preparation to overcome this issue.

The vascular endothelial growth factor A (*VEGFA*) gene plays an important role in angiogenesis. It has been shown that stress-induced *VEGFA* provides potent chemoattractant signals not only for endothelial cells but also for osteoclasts and stem cells to establish appropriate vascularization associated with bone formation [63]. Also, *VEGFA* is closely involved in bone healing [64].

While the *TNFRSF11B/OPG* gene is crucial in maintaining bone density by regulating the activity of osteoclasts, the exposure to 1 h FSS did not affect its gene expression herein, which might be caused by the rather short duration of stress application [65,66].

#### 4.5.3. Effect of FSS on Inflammation

The current study analyzed the effect of FSS on the expression of various inflammation-related genes in hPDLCs, i.e., *IL6*, *CXCL8/IL8*, and *PTGS2/COX2*. Mechanical stimulation with FSS caused significant upregulation of *PTGS2/COX2* in the cultures of cells of both donors but only in cells of donor 2 when considering *IL6*.

*PTGS2* encodes the prostaglandin-endoperoxide synthase 2 (also known as cyclooxygenase-2, *COX2*), which is centrally involved in the prostaglandin pathway. Prostaglandins are lipid compounds with a wide array of biological effects in the human body, including inflammation and bone metabolism affecting both, osteoclasts and osteoblasts [67,68]. It is commonly accepted that prostaglandins play an important role in bone resorption during inflammation and immobilization [69]. Moreover, the inhibition of the prostaglandins in vivo using flurbiprofen has been found to reduce the number of osteoclasts in bone and thus decrease tooth movement [70]. Previous reports have suggested that FSS upregulates *FOS* and *PTGS2*/*COX2* expression in osteoblasts resulting in stronger differentiation, and proliferation of osteoblasts, more intensive bone calcification, improved fracture healing, and ultimately stronger bone formation [71,72]. Also, *PTGS2/COX2* contains an AP-1 binding site in its promoter [73], indicating that the expression of this gene might be regulated by *FOS*. The activation of the AP-1 pathway plays an important role in osteoblast proliferation and differentiation [73].

The Western blot data show an increase in PTGS2/COX2 in FSS-treated cells compared to their controls. The enzymatically active form of PTGS2/COX2 has a molecular weight of ~70 kDa and has been considered difficult to identify due to its banding pattern in Western blots [74]. Previous research suggests the necessity for RT-qPCR data and positive control to confirm the observation of PTGS2/COX2 using Western blot [74]. Using a positive control confirms that the Western blot procedure and the antibodies used therein are functioning correctly, regardless of whether the test samples yield negative results. This also helps to ensure that any negative findings are reliable. Therefore, we showed that 6 dyn/cm^2^ for 1 h resulted in the upregulation of PTGS2/COX2 protein expression compared to the control, with a greater difference being shown in donor 2 compared to donor 1.

The pro-inflammatory cytokine interleukin-8 (IL8) primarily acts as a chemokine (*CXCL8*), activating and attracting immune cells, particularly neutrophils to sites of infection or injury. An increased level of IL8 in the gingival crevicular fluid has been found during OTM, which was interpreted as an evident response to clinical orthodontic load [75]. IL8 regulates bone resorption by modulating the expression of the osteoclastogenic factor RANKL in osteoblasts [76]. IL8 is not limited to inflammation but has also been found to be involved in distinct tissue-forming processes. Among others, IL8 might function as an endothelial cell growth and survival factor, enhancing the proliferation of endothelial cells through the expression of antiapoptotic genes and activation of *MMP2* and *MMP9* [77]. Moreover, IL8 upregulates the expression of the *VEGFA* gene through the *NF-κB* pathway [78].

Interleukin-6 (IL6) primarily acts as a proinflammatory signaling molecule and plays a role in various physiological processes including PDL and bone turnover [79]. It has been found that IL6 may affect bone mass in vivo by influencing osteocytes signaling towards osteoblasts [80]. Moreover, IL6 is involved in the activation and proliferation of osteoclasts in vitro [81]. Due to the complex multi-functional catabolic and anabolic roles of *IL6*, *CXCL8/IL8*, and *PTGS2/COX2* and the partially conflicting data, it can be assumed that these genetic loci regulate bone/tissue remodeling during OTM by maintaining a dynamic equilibrium between tissue formation and resorption.

#### 4.5.4. Heterogeneity Between Donors and Fluid Shear Stress

Although the hPDLCs used in this study were isolated and cultivated using standardized protocols, they were isolated from donors of similar age but different sex (14-year female vs. 16-year male). Inherent donor-related differences such as sex and age represent confounding variables that might systematically influence gene expression [2]. Pooling cells of multiple donors is a technique used to minimize variability and reduce the influence of individual differences, thereby providing an average cell response [82]. However, this would require much more donors to address this issue.

The differences in gene expression between the cells of both donors might be attributed to various reasons. Among others, an age-dependent progressive thinning of the periodontal ligament, accompanied by a decrease in mitotic activity and soluble collagen levels has been suggested [83]. Due to the close age of the two donors, this reason might not be responsible for the differences in gene expression.

Apart from the tooth support, the periodontal ligament can adapt to mechanical forces. Mechanical loads that act on cells during OTM may influence the orientation of PDL fibroblasts, the cell morphology, and the synthesis of proteins, i.e., as collagen type I, type XII, and fibronectin [43,84]. Moreover, the regulation of specific genes may also influence the periodontal fibroblasts during OTM and thus alter their behavior [44]. In addition, the loss of normal occlusal forces due to malocclusion or as a result of certain orthodontic appliances may lead to atrophic changes such as narrowing of the PDL space, disorientation, reduction in size and number of collagen fibers, and vascular impairment [85,86]. Therefore, orthodontic pretreatment might induce persistent changes in the periodontal ligament cells. While this may partly explain the considerably different mechanical thresholds to mechanical stress in cultures from donor 1 compared to donor 2, other factors, such as sex or age, must also be considered and should be clarified in further studies.

We recognize inter-donor variation in this study due to the small sample size. It is recommended for further studies to include more than two donors to increase the sample size, while ensuring similar age, sex, passage number, cell number, and health status [21].

### 4.6. Strengths and Limitations

Herein, we described the construction and usage of a custom-made fluid flow system to apply FSS to cells of the periodontium. Several strengths and limitations of the study and chamber design were concluded as follows:

#### 4.6.1. Strengths

- Biocompatibility.
- Durability.
- Flexibility (can be adapted to different research questions).
- Decomposability (can be disassembled for sample collection).
- Large sample for further gene/intracellular protein analysis.
- Affordability (cost-effective).

#### 4.6.2. Limitations

- Using a large volume of cell-culturing media (diluted supernatant).
- Technique sensitive during assembly and disassembly
- Reusability requires further steps afterward for disinfection and sterilization.
- The preparation of the experimental setup is completed outside the incubator.
- To account for biological variability, additional donors should be included.
- The flow chamber is not compatible with live microscopy, making it difficult to investigate potential regions of turbulence using a tracer dye or real-time cellular/molecular visualization.
- Only a few genes were included in this study, which may not fully reflect the complete biological picture related to FSS.

## 5. Conclusions

The fluid flow apparatus presented herein has shown durability and compatibility while maintaining a controlled dynamic condition. The application of FSS for 1 h resulted in the upregulation of genes responsible for regeneration and inflammation in hPDLCs despite showing considerable differences between both donors. Therefore, FSS is suggested to have an inflammatory and regenerative impact during OTM on the periodontal tissues. More sophisticated experimental models might be employed to achieve further information on the effects of FSS on PDL cells during OTM.

## Figures and Tables

**Table 1:** Individual components of the FSS system. The part numbers refer to the individually numbered parts in Figure 3B.

Part No.	Part	Type	Source
1	Heating water bath	Lauda Aqualine AL5	Lauda, Lauda-Königshofen, Germany
2	Reservoir and multiple distributors for bottles GL 45 with connectors	PY86.1	Carl Roth, Karlsruhe, Germany
3	Peristaltic pump w/2 pump head (3 rolls)	LabV3 with YZ151x PPS pump heads	Drifton A/S, Hvidovre, Denmark
4	Constant flow pulse damper	D1606-6B-PSU	SCPOGO LABS, Beijing, China
5a	Bubble trap consisting of barbed T-connector	FESTO T-PK-4, 9585	Landefeld Druckluft und Hydraulik GmbH, Kassel, Germany
5b	Stainless-steel lever air control valve	L × W × H: 30 × 21 × 8 mm^3^	Sourcing map; url: https://sourcingmap.com (accessed on 21 October 2024)
6	Chamber	See above	
7	Screw clamps	Wisent Laubsägezwinge	Hornbach, Munich, Germany
8	Sterile silicone tubing	Longer BioSilicone (WT 1.6 mm, ID 4.8 mm, OD 8.0 mm	Drifton A/S, Hvidovre, Denmark
	Microscopy slide (with cells seeded in a specified area)	Epredia™ Microscope Slides, Cut, 1mm (AA00000102E01MNZ10)	New Erie Scientific LLC, Portsmouth, NH, USA

**Table 2:** Details of the PCR primers used for gene quantification (more details in Supplement File S1).

Gene	GenBank Accession Number	Primer Sequence (f:5-Forward Primer-3; r:5-Reverse Primer-3)	Anneal. Temp. (°C)	Amplicon Length (bp)	Primer Efficiency
*RUNX2*	NM_001015051.4	f: GCGCATTCCTCATCCCAGTA r: GGCTCAGGTAGGAGGGGTAA	58	176	2.033
*IL6*	NM_000600.5	f: TGGCAGAAAACAACCTGAACC r: TGGCTTGTTCCTCACTACTCTC	58	168	1.931
*PTGS2/COX2*	NM_000963.4	f: AAGCCTTCTCTAACCTCTCC r: GCCCTCGCTTATGATCTGTC	58	234	1.988
*FOS*	NM_005252.4	f: GCTTTGCAGACCGAGATTGC r: TTGAGGAGAGGCAGGGTGAA	58	203	1.942
*SP7*	NM_001173467.3	f: GGCACAAAGAAGCCGTACTC r: CACTGGGCAGACAGTCAGAA	61	247	2.077
*TNFRSF11B*	NM_002546.4	f: TCAAGCAGGAGTGCAATCG r: AGAATGCCTCCTCACACAGG	60	342	1.972
*VEGFA*	NM_001317010.2	f: GCTGTCTTGGGTGCATTGGA r: ATGATTCTGCCCTCCTCCTTCT	58	100	2.071
*CXCL8/IL8*	NM_001354840.3	f: CAGAGACAGCAGAGCACACAA r: TTAGCACTCCTTGGCAAAAC	55	170	1.948
*RPL0*	NM_001002.4	f: GAAACTCTGCATTCTCGCTTCC r: GACTCGTTTGTACCCGTTGATG	64	120	1.988
*RPL22*	NM_000983.4	f: TGATTGCACCCACCCTGTAG r: GGTTCCCAGCTTTTCCGTTC	61	98	2.055

**Table 3:** Summary statistics comparing the impact of FSS on the expression of specific genes (*FOS*, *PTGS2/COX2*, *CXCL8*/*IL8*, *IL6*, *RUNX2*, *SP7*, *TNFRSF11B*, and *VEGFA*), represented as fold change, in hPDLCs. The gene expression data is reported as fold change (2^−ΔΔCq^) and presented as mean values, standard deviation (SD), median, minimum (min), and maximum (max). *p*-values were determined using the Mann-Whitney U Test (U-test).

Gene		Control					FSS					U-Test	
		Mean	SD	Min	Max	Median	Mean	SD	Min	Max	Median	*p*	Sig.^†^
*CXCL8*	Donor 1 (N = 4)	1.00	0.10	0.89	1.13	1.00	1.33	0.15	1.14	1.50	1.33	0.029	*
	Donor 2 (N = 4)	1.06	0.42	0.63	1.59	1.01	1.53	0.14	1.44	1.74	1.47	0.200	n.s.
	Total (N = 8)	1.03	0.28	0.63	1.59	1.00	1.43	0.17	1.14	1.74	1.45	0.010	*
*FOS*	Donor 1 (N = 4)	1.01	0.18	0.84	1.18	1.01	2.00	0.45	1.71	2.67	1.81	0.029	*
	Donor 2 (N = 4)	1.13	0.62	0.52	1.92	1.04	3.37	1.02	2.21	4.44	3.41	0.029	*
	Total (N = 8)	1.07	0.43	0.52	1.92	1.01	2.68	1.03	1.71	4.44	2.44	0.001	**
*IL6*	Donor 1 (N = 4)	1.04	0.32	0.69	1.45	1.01	1.03	0.53	0.67	1.80	0.82	0.886	n.s.
	Donor 2 (N = 4)	1.00	0.04	0.95	1.05	1.00	1.60	0.84	1.10	2.85	1.22	0.029	*
	Total (N = 8)	1.02	0.21	0.69	1.45	1.00	1.31	0.72	0.67	2.85	1.11	0.505	n.s.
*PTGS2*	Donor 1 (N = 4)	1.02	0.24	0.77	1.30	1.01	1.87	0.14	1.73	2.01	1.87	0.029	*
	Donor 2 (N = 4)	1.02	0.26	0.74	1.36	1.00	2.69	0.66	2.01	3.34	2.71	0.029	*
	Total (N = 8)	1.02	0.23	0.74	1.36	1.00	2.28	0.62	1.73	3.34	2.01	<0.001	***
*RUNX2*	Donor 1 (N = 4)	1.03	0.26	0.74	1.36	1.00	1.31	0.17	1.19	1.55	1.24	0.200	n.s.
	Donor 2 (N = 4)	1.01	0.18	0.84	1.19	1.01	1.70	0.53	1.21	2.26	1.67	0.029	*
	Total (N = 8)	1.02	0.21	0.74	1.36	1.00	1.50	0.42	1.19	2.26	1.28	0.005	**
*SP7*	Donor 1 (N = 4)	1.01	0.15	0.85	1.18	1.00	1.47	0.67	0.80	2.32	1.38	0.486	n.s.
	Donor 2 (N = 4)	1.12	0.50	0.58	1.75	1.09	15.47	24.05	0.23	51.28	5.17	0.343	n.s.
	Total (N = 8)	1.07	0.35	0.58	1.75	1.00	8.47	17.44	0.23	51.28	1.99	0.195	n.s.
*TNFRSF11B*	Donor 1 (N = 4)	1.00	0.10	0.89	1.12	1.00	1.05	0.06	0.99	1.12	1.05	0.486	n.s.
	Donor 2 (N = 4)	1.02	0.22	0.77	1.30	1.01	1.09	0.26	0.73	1.35	1.13	0.686	n.s.
	Total (N = 8)	1.01	0.16	0.77	1.30	1.01	1.07	0.18	0.73	1.35	1.08	0.328	n.s.
*VEGFA*	Donor 1 (N = 4)	1.00	0.07	0.92	1.09	1.00	1.17	0.10	1.08	1.28	1.16	0.114	n.s.
	Donor 2 (N = 4)	1.00	0.11	0.89	1.13	1.00	1.33	0.34	0.95	1.68	1.34	0.200	n.s.
	Total (N = 8)	1.00	0.08	0.89	1.13	1.00	1.25	0.25	0.95	1.68	1.19	0.021	*

^†^ Significance (Sig.) levels were: *, *p* < 0.05; **, *p* < 0.01; ***, *p* < 0.001; n.s., not significant.

## Data Availability

All data are presented in the manuscript. Please contact the corresponding authors for additional information.

## Supplementary Materials

The following supporting information can be downloaded at: https://www.mdpi.com/article/10.3390/cells13211751/s1, Supplementary File S1: MIQE report, primer evaluation, and primer-specific qPCR settings; Supplementary File S2: RefFinder evaluation of reference genes; Supplementary File S3: Western blot data.

## List of Abbreviations

3D	Three dimensional
CAD	Computer-aided design
CFD	Computational fluid dynamics
CXCL8	C-X-C Motif Chemokine Ligand 8 (also known as interleukin 8, IL-8)
FC	Fold change
FOS	Proto-oncogene Fos; subunit of the AP-1 transcription factor
FSS	Fluid shear stress
hPDLCs	Human periodontal ligament cells
IL6	Interleukin-6
MIQE	“Minimum Information for Publication of Quantitative Real-Time PCR Experiments”
OTM	Orthodontic tooth movement
PDL	Periodontal ligament
PTGS2	Prostaglandin-endoperoxide synthase (also known as cyclooxygenase-2, COX-2)
RUNX2	Runt-related transcription factor 2
SP7	Sp7 transcription factor (also known as osterix, OSX)
TNFRSF11B	TNF receptor superfamily member 11b (also known as osteoprotegerin, OPG)
VEGFA	Vascular endothelial growth factor A
