# Supplementary material for: Development of a Custom Fluid Flow Chamber for Investigating the Effects of Shear Stress on Periodontal Ligament Cells
Source: Cells. 2024 Oct 23;13(21):1751. doi: 10.3390/cells13211751 (PMC11545369; doi:10.3390/cells13211751)
Supplement: Supplementary file 1 [file cells-13-01751-s001.zip › Supplementary File S1.pdf]

Supplementary File S1: MIQE report, primer evaluation, and primer-specific qPCR settings.

To the manuscript:

"Development of a Custom Fluid Flow Chamber for Investigating the Effects of Shear Stress on Periodontal Ligament Cells"

Contents

Supplementary Table S1.1: MIQE checklist 2
Supplementary Table S1.2: Primer validation by RT-qPCR 5
Supplementary Table S1.3: Primer evaluation and primer-specific qPCR settings 7
References....................7

Supplementary Table S1.1: MIQE checklist

Reference: Bustin et al. (2010). BMC Mol Biol; 11:74.

	Details	Checklist
**Sample/Template**		
Source	If cancer, was biopsy screened for adjacent normal tissue?	Human alveolar-bone derived osteoblasts (hOBs) were obtained anonymously from a male donor undergoing a surgical procedure based orthodontic treatment and isolated according to established procedures (Ng and Schantz 2010; Shi et al. 2019a; Shi et al. 2019b).
Method of preservation	Liquid N2/RNAlater/formalin	Cell lysates were snap frozen in liquid N2 and stored at -80°C until all samples were collected.
Storage time (if appropriate)	If using samples >6 months old	Not applicable.
Handling	Fresh/frozen/formalin	Cell lysates were prepared using RNA lysis buffer from Quick-RNA^{TM} MicroPrep kit (R1051; Zymo). They were snap-frozen in liquid nitrogen, and then stored at -80°C until further use for RNA extraction.
Extraction method	TriZol/columns	Defrosted cells lysates were passed through QIAshredder™ columns (Qiagen) to shear genomic DNA. The Quick-RNA™ Miniprep Kit (Zymo) was used for further RNA purification. After primary column purification, DNase I digestion was applied to reduce genomic DNA contamination as described by the manufacturer (Zymo). Finally, DNase/RNase-free water was used to elute the RNA from the columns. Before storage in the -80°C, RNase inhibitor RNasin® (Promega) was added to each preparation.
RNA:DNA-free	Intron-spanning primers/no RT control	Most primers were intron-spanning (Supplementary Table 1.2). Treatment with QIAshredder™ columns (Qiagen) and DNase I (Zymo) digestion were applied to reduce genomic DNA contamination. RT- (no RT) controls were tested and showed no contamination of genomic DNA.
Concentration	Nanodrop/ribogreen/microfluidics	Purity and concentration of extracted RNA were detected photometrically (Nanodrop ND-1000; PeqLab). Ratio of $A_{260/280}$>1.8 was found, indicating free of protein contamination during RNA preparations.
RNA: integrity	Microfluidics/3':5' assay	No.
Inhibition-free	Method of testing	Serial dilution of cDNA as shown in “Primer efficiency” in Supplementary Table S1.2 below.
**Assay optimisation/validation**		
Accession number	RefSeq XX_1234567	Table 2 in the manuscript and Supplementary Table S1.3. All others were previously published.
Amplicon details	Exon location, amplicon size	Supplementary Table S1.3. All others were previously published.
Primer sequence	Even if previously published	Table 2 in the manuscript; Supplementary Tables S1.2 and S1.3.
*Probe sequence**	Identify LNA or other substitutions	No probes were used.
In silico	BLAST/Primer-BLAST/m-fold	Primer-BLAST, UCSC In-Silico PCR, and ENSEMBL were used for *in silico* testing.

	Details	Checklist
empirical	Primer concentration/annealing temperature	The optimal annealing temperatures were first identified by gradient PCR (TProfessional Gradient; Biometra, Goettingen, Germany) and then were finalized by qPCR on Roche LightCycler® 480 (LC480). Optimal annealing temperatures are recorded in Supplementary Table S1.2 below.
Priming conditions	Oligo-dT/random/combination/target-specific	The SuperScript® IV First Strand Synthesis System (Invitrogen) was used for cDNA synthesis with random hexamers provided. For each cDNA synthesis reaction, 600 ng total RNA was used. Target-specific primers for qPCR were used after assessment (Table 2 of the manuscript; Supplementary Table S1.3).
PCR efficiency	Dilution curve	Information on serial dilutions and primer efficiency was summarized in Supplementary Table S1.2. For each gene, two technical replicates were used for each dilution for qPCR. For analysis of qPCR including the standard curves, LC480 software version 1.5.0.39 was used.
Linear dynamic range	Spanning unknown targets	The analyzing software for qPCR appointed the linear dynamic range automatically.
Limits of detection	LOD detection/accurate quantification	The analyzing software for qPCR appointed the LOD automatically.
Intra-assay variation	Copy numbers not Cq	Each gene was detected on one individual plate.
**RT/PCR**		
Protocols	Detailed description, concentrations, volumes	For real-time PCR, LightCycler® 480 SYBR Green I Master kit (04887352001; Roche Diagnostics GmbH, Mannheim, Germany) was used to detect gene expression of *PTGS2/COX2, IL6, CXCL8/IL8, RUNX2, FOS, VEGFA, SP7, and TNFRSF11B/OPG* and using the LightCycler® 480 with LC480 software version 1.5.0.39 (both from Roche Molecular Diagnostics, Basel, Switzerland). According to the manufacturer' protocol, 5 µl diluted cDNA (1:10 with double distilled, sterile water), 1 µl gene-specific forward primer, 1 µl gene-specific reverse primers, 3 µl PCR water and 10 µl qPCR mastermix were added for reaction. PCR reactions proceeded as follows: 10 min of initial denaturation at 95 °C and 45 cycles of amplifications. Each amplification consisted of three steps: 15 s of denaturation at 95 °C, 15 s of specific annealing temperature for each primer pair and 15 s of elongation at 72 °C. For each plate, both no template controls (NTC) and no RT (reverse transcriptase) controls were added. NTCs were included for detection of primer dimers, while no RT controls were assessed for genomic DNA contamination.
Reagents	Supplier, Lot number	Primers for genes were synthesized using sequences from related literature. The primers were verified by *in silico* tests using related bioinformatic tools given in Supplementary Table S1.2. All primers were synthesized by TIB Molbiol Syntheselabor GmbH (Berlin, Germany). Information on the kits used (Quick-RNA™ MicroPrep kit; SuperScript® IV First Strand Synthesis kit, Invitrogen; LightCycler® 480 SYBR Green I Master kit, Roche) were all given in the manuscript.
Duplicate RT	ΔCq	No, but two technical replicates were repeated for each biological replicate at minimum.
NTC	Cq & melt curves	Yes
NAC	ΔCq beginning:end of qPCR	No, as no probes were used.
Positive control	Inter-run calibrators	No, each gene was tested on one plate with all samples included.
**Data analysis**		

	Details	Checklist
Specialist software	e.g., QBAsePlus	IBM SPSS Statistics 29 (IBM Corp., Armonk, NY, USA)
Statistical justification	e.g., biological replicates	For each force magnitude for every force duration, three biological replicates were used. Each biological replicate was repeated with two technical replicates, giving a total of 6 amplifications of qPCR.
Transparent, validated normalization	e.g., GeNorm summary	After testing with RT-qPCR using cDNA from some samples and assessment with RefFinder, *RPL0* and *RPL22* were proved to be most stable in this experiment among the panel of reference genes. Therefore, *RPL0* and *RPL22* were used as reference genes for following analysis.

Supplementary Table S1.2: Primer validation by RT-qPCR

Gene symbol	GenBank Accession Number	Primer sequence (f: 5'-forward primer-3'; r: 5'-reverse primer-3')	Source of primer sequence	Prediluted cDNA (1:10) used	Specificity by melting curve / T_m (°C)	Specificity by agarose gel / amplicon size (bp)	Annealing temp. (°C)	Dilution series used for efficiency testing	Primer efficiency			
									Efficiency	Error	Slope	Y Intercept
**Genes of interest**<br>Previously published												
RUNX2	NM_001015051.4	f: GCGCATTCCTCATCCCAGTA<br>r: GGCTCAGGTAGGAGGGGTAA	(Janjic Rankovic et al. 2020; Shi et al. 2019a; Sun et al. 2022)	Prediluted	Yes / 83	Yes / 176	58	Predil., 1:2, 1:4, 1:8, 1:16	2.033	0.00791	3.245	29.58
IL6	NM_000600.5	f: TGGCAGAAAACAACCTGAACC<br>r: TGGCTTGTTCCTCACTACTCTC	(Janjic Rankovic et al. 2020; Shi et al. 2019a; Sun et al. 2022)	Prediluted	Yes / 77.8	Yes / 168	58	Predil., 1:10, 1:100, 1:1000, 1;10,000	1.931	0.00966	-3.499	35.19
PTGS2/COX2	NM_000963.4	f: AAGCCTTCTCTAACCTCTCC<br>r: GCCCTCGCTTATGATCTGTC	(Janjic Rankovic et al. 2020; Shi et al. 2019a; Sun et al. 2022)	Prediluted	Yes /79	Yes / 234	58	Predil., 1:10, 1:100, 1:1000, 1;10,000	1.988	0.0107	-3.350	34.19
FOS	NM_005252.4	f: GCTTTGCAGACCGAGATTGC<br>r: TTGAGGAGAGGCAGGGTGAA	(Janjic Rankovic et al. 2020; Sun et al. 2022)	Prediluted	Yes / 85	Yes / 203	58	Predil., 1:2, 1:4, 1:8, 1:16	1.942	0,00478	-3,470	27,72
SP7	NM_001173467.3	f: GGCACAAAGAAGCCGTACTC<br>r: CACTGGGCAGACAGTCAGAA	(Gronthos et al. 2003; Sun et al. 2022)	Prediluted	Yes / 83.9	Yes / 247	61	Predil., 1:2, 1:4, 1:8, 1:16	2.077	0.0146	-3.151	35.47
TNFRSF11B	NM_002546.4	f: TCAAGCAGGAGTGCAATCG<br>r: AGAATGCCTCCTCACACAGG	(Sun et al. 2022; Yang et al. 2010)	Prediluted	Yes / 83	Yes / 342	60	Predil., 1:10, 1:100, 1:1000, 1;10,000	1.972	0.0240	-3.391	39.26
**Included in this study**												
VEGFA	NM_001317010.2	f: GCTGTCTTGGGTGCATTGGA<br>r: ATGATTCTGCCCTCCTCCTTCT	(Chae et al. 2011)	Prediluted	Yes /83.8	Yes / 100	58	Predil., 1:10, 1:100, 1:1000, 1;10,000	2.071	0.0220	-3.164	36.52
CXCL8/IL8	NM_001354840.3	f: CAGAGACAGCAGAGCACACAA<br>r: TTAGCACTCCTTGGCAAAAC	(Li et al. 2007)	Prediluted	Yes /81.9	Yes / 170	55	Predil., 1:10, 1:100, 1:1000, 1;10,000	1.948	0.0114	-3.452	36.54
**Reference genes**												

Gene symbol	GenBank Accession Number	Primer sequence (f: 5'-forward primer-3'; r: 5'-reverse primer-3')	Source of primer sequence	Prediluted cDNA (1:10) used	Specificity by melting curve / T_m (°C)	Specificity by agarose gel / amplicon size (bp)	Annealing temp. (°C)	Dilution series used for efficiency testing	Primer efficiency			
									Efficiency	Error	Slope	Y Intercept
EEF1A1	NM_001402.6	f: CCTGCCTCTCCAGGATGTCTAC<br>r: GGAGCAAAGGTGACCACCATAC	(Nazet et al. 2020)	Prediluted	Yes / 79	Yes /105	61	Predil., 1:10, 1:100, 1:1000, 1;10,000	2.005	0.0194	-3.311	32.18
GAPDH	NM_002046.7	f: CTCCTGTTCGACAGTCAGCC<br>r: CGACCAAATCCGTTGACTCC	(Nazet et al. 2020; Sun et al. 2022)	Prediluted	Yes / 81.5	Yes / 103	52	Predil., 1:10, 1:100, 1:1000, 1;10,000	1.955	0.00797	-3.433	34.32
POLR2A	NM_000937.5	f: TCGCTTACTGTCTTCCTGTTGG<br>r: TGTGTTGGCAGTCACCTTCC	(Nazet et al. 2020; Sun et al. 2022)	Prediluted	Yes / 81	Yes / 108	58	Predil., 1:10, 1:100, 1:1000, 1;10,000	2.072	0.0193	-3.161	37.74
PPIB	NM_000942.5	f: TTCCATCGTGTAATCAAGGACTTC<br>r: GCTCACCGTAGATGCTCTTTC	(Nazet et al. 2020; Sun et al. 2022)	Prediluted	Yes / 79	Yes / 88	55	Predil., 1:10, 1:100, 1:1000, 1;10,000	1.931	0.0115	-3.500	37.08
RNA18S5	NR_003286.4	f: AACTGCGAATGGCTCATTAAATC<br>r: GCCCGTCGGCATGTATTAG	(Nazet et al. 2020; Sun et al. 2022)	Prediluted	Yes / 77	Yes / 103	55	Predil., 1:10, 1:100, 1:1000, 1;10,000	1.991	0.00498	-3.344	22.85
RPL0	NM_001002.4	f: GAAACTCTGCATTCTCGCTTCC<br>r: GACTCGTTTGTACCCGTTGATG	(Nazet et al. 2020; Sun et al. 2022)	Prediluted	Yes /80.5	Yes /120	64	Predil., 1:10, 1:100, 1:1000, 1;10,000	1.988	0.00679	-3.351	34.57
RPL22	NM_000983.4	f: TGATTGCACCCACCCTGTAG<br>r: GGTTCCCAGCTTTTCCGTTC	(Nazet et al. 2020; Sun et al. 2022)	Prediluted	Yes / 76.5	Yes / 98	61	Predil., 1:10, 1:100, 1:1000, 1;10,000	2.055	0.00931	-3.196	35.50
YWHAZ	NM_003406.4	f: AGGAGATTACTACCGTTACTTGGC<br>r: AGCTTCTTGGTATGCTTGTTGTG	(Nazet et al. 2020; Sun et al. 2022)	Prediluted	Yes / 77.8	Yes / 91	55	Predil., 1:10, 1:100, 1:1000, 1;10,000	1.862	0.00937	-3.705	38.69

Supplementary Table S1.3: Primer evaluation and primer-specific qPCR settings

Reference	Gene Name	Primer sequence (f: 5'-forward primer-3'; r: 5'-reverse primer-3')	Primer-BLAST (bp)		T_m (°)		Splice variants	*In silico* PCR specificity	*Annealing Temperature (°C)*	*Tm (°C)*	*4th Step (°C)*
			mRNA	Genomic	For.	Rev.					
(Chae et al. 2011)	*VEGFA*	F: GCTGTCTTGGGTGCATTGGA<br>R: ATGATTCTGCCCTCCTCCTTCT	100	3,128	60.9	60.6	yes	Yes	58	83.7	81
(Li et al. 2007)	*IL8*	F: CAGAGACAGCAGAGCACACAA<br>R: TTAGCACTCCTTGGCAAAAC	170	989	60.54	56.53	989bp for IL8-isoforms 1+2 precursors; 104184 also with mismatches in the reverse primer	Yes	55	82.5	80

References

Chae HS, Park HJ, Hwang HR, Kwon A, Lim WH, Yi WJ, Han DH, Kim YH, Baek JH (2011). The effect of antioxidants on the production of pro-inflammatory cytokines and orthodontic tooth movement. Mol Cells; 32(2):189-96.

Gronthos S, Zannettino AC, Hay SJ, Shi S, Graves SE, Kortesidis A, Simmons PJ (2003). Molecular and cellular characterisation of highly purified stromal stem cells derived from human bone marrow. J Cell Sci; 116(Pt 9):1827-35.

Janjic Rankovic M, Docheva D, Wichelhaus A, Baumert U (2020). Effect of static compressive force on in vitro cultured PDL fibroblasts: monitoring of viability and gene expression over 6 days. Clin Oral Investig; 24(7):2497-2511.

Li L, Redding S, Dongari-Bagtzoglou A (2007). Candida glabrata: an emerging oral opportunistic pathogen. J Dent Res; 86(3):204-15.

Nazet U, Schroder A, Spanier G, Wolf M, Proff P, Kirschneck C (2020). Simplified method for applying static isotropic tensile strain in cell culture experiments with identification of valid RT-qPCR reference genes for PDL fibroblasts. Eur J Orthod; 42(4):359-370.

Ng KW, Schantz J-T (2010). A Manual for Primary Human Cell Culture. 2nd ed. New Jersey: World Scientific.

Shi J, Baumert U, Folwaczny M, Wichelhaus A (2019a). Influence of static forces on the expression of selected parameters of inflammation in periodontal ligament cells and alveolar bone cells in a co-culture in vitro model. Clin Oral Investig; 23(6):2617-2628.

Shi J, Folwaczny M, Wichelhaus A, Baumert U (2019b). Differences in RUNX2 and P2RX7 gene expression between mono- and coculture of human periodontal ligament cells and human osteoblasts under compressive force application. Orthod Craniofac Res; 22(3):168-176.

Sun C, Janjic Rankovic M, Folwaczny M, Stocker T, Otto S, Wichelhaus A, Baumert U (2022). Effect of Different Parameters of In Vitro Static Tensile Strain on Human Periodontal Ligament Cells Simulating the Tension Side of Orthodontic Tooth Movement. Int J Mol Sci; 23(3):1525.

Yang Y, Yang Y, Li X, Cui L, Fu M, Rabie AB, Zhang D (2010). Functional analysis of core binding factor a1 and its relationship with related genes expressed by human periodontal ligament cells exposed to mechanical stress. Eur J Orthod; 32(6):698-705.
