# Supplementary material for: Development of a Custom Fluid Flow Chamber for Investigating the Effects of Shear Stress on Periodontal Ligament Cells
Source: Cells. 2024 Oct 23;13(21):1751. doi: 10.3390/cells13211751 (PMC11545369; doi:10.3390/cells13211751)

Supplementary File S2: RefFinder evaluation of reference genes

To the manuscript:

"Investigating the influence of fluid flow shear stress on periodontal ligament cells through a custom-made fluid flow chamber"

RefFinder @ https://www.ciidirsinaloa.com.mx/RefFinder-master/

Citation: Xie, Xiao, Chen, Xu, Zhang (2012). miRDeepFinder: a miRNA analysis tool for deep sequencing of plant small RNAs. Plant Mol. Biol. 80 (1), 75-84.

References

- *BestKeeper*: Pfaffl MW, Tichopad A, Prgomet C, Neuvians TP. 2004. Determination of stable housekeeping genes, differentially regulated target genes and sample integrity: BestKeeper--Excel-based tool using pair-wise correlations. Biotechnology letters 26:509-515.
- *NormFinder*: Andersen CL, Jensen JL, Orntoft TF. 2004. Normalization of real-time quantitative reverse transcription-PCR data: a model-based variance estimation approach to identify genes suited for normalization, applied to bladder and colon cancer data sets. Cancer research 64:5245-5250.
- *Genorm*: Vandesompele J, De Preter K, Pattyn F, Poppe B, Van Roy N, De Paepe A, Speleman F. 2002. Accurate normalization of real-time quantitative RT-PCR data by geometric averaging of multiple internal control genes. Genome biology 3:RESEARCH0034.
- *The comparative delta-Ct method*: Silver N, Best S, Jiang J, Thein SL. 2006. Selection of housekeeping genes for gene expression studies in human reticulocytes using real-time PCR. BMC molecular biology 7:33.

Contents

Raw data.....2

RefFinder.....3

Summary.....3

Comprehensive Ranking.....3

Delta CT.....4

BestKeeper.....5

normFinder.....6

Genorm.....7

Raw data

Condition	Sample	Replicate	RPL0	EEF1A1	RPL22	PPIB	RNA18s5	YWHAZ	POLR2A	GAPDH
FSS	1	1	22.98	18.97	22.71	24.35	9.19	23.00	23.93	20.97
FSS	1	2	22.93	18.94	22.74	24.32	9.27	22.90	23.86	20.77
FSS	2	1	22.74	18.75	22.82	24.01	8.93	22.81	23.79	20.64
FSS	2	2	22.77	18.82	22.76	24.11	8.99	22.90	23.83	20.52
Ctrl	1	1	22.76	18.87	22.73	23.96	9.37	23.10	24.25	20.77
Ctrl	1	2	22.77	18.90	22.68	23.98	9.36	23.11	24.24	20.80
Ctrl	2	1	23.22	19.45	22.95	24.09	9.55	23.64	23.83	20.89
Ctrl	2	2	23.22	19.49	22.95	24.11	9.64	23.63	23.89	20.84

	Ctrl (N=4)	FSS (N=4)	All (N=8)
EEF1A1			
Mean (SD)	19.2 (0.338)	18.9 (0.103)	19.0 (0.284)
Median [Min, Max]	19.2 [18.9, 19.5]	18.9 [18.8, 19.0]	18.9 [18.8, 19.5]
GAPDH			
Mean (SD)	20.8 (0.0520)	20.7 (0.193)	20.8 (0.141)
Median [Min, Max]	20.8 [20.8, 20.9]	20.7 [20.5, 21.0]	20.8 [20.5, 21.0]
POLR2A			
Mean (SD)	24.1 (0.224)	23.9 (0.0591)	24.0 (0.185)
Median [Min, Max]	24.1 [23.8, 24.3]	23.8 [23.8, 23.9]	23.9 [23.8, 24.3]
PPIB			
Mean (SD)	24.0 (0.0759)	24.2 (0.164)	24.1 (0.147)
Median [Min, Max]	24.0 [24.0, 24.1]	24.2 [24.0, 24.4]	24.1 [24.0, 24.4]
RNA18S5			
Mean (SD)	9.48 (0.138)	9.10 (0.161)	9.29 (0.248)
Median [Min, Max]	9.46 [9.36, 9.64]	9.09 [8.93, 9.27]	9.32 [8.93, 9.64]
RPL0			
Mean (SD)	23.0 (0.263)	22.9 (0.118)	22.9 (0.202)
Median [Min, Max]	23.0 [22.8, 23.2]	22.9 [22.7, 23.0]	22.9 [22.7, 23.2]
RPL22			
Mean (SD)	22.8 (0.143)	22.8 (0.0465)	22.8 (0.105)
Median [Min, Max]	22.8 [22.7, 23.0]	22.8 [22.7, 22.8]	22.8 [22.7, 23.0]
YWHAZ			
Mean (SD)	23.4 (0.306)	22.9 (0.0776)	23.1 (0.324)
Median [Min, Max]	23.4 [23.1, 23.6]	22.9 [22.8, 23.0]	23.1 [22.8, 23.6]

RefFinder

Summary

	Ranking Order (Better--Good--Average)							
**Method**	**1**	**2**	**3**	**4**	**5**	**6**	**7**	**8**
Delta CT	RPL0	GAPDH	RPL22	RNA18s5	EEF1A1	YWHAZ	PPIB	POLR2A
BestKeeper	RPL22	GAPDH	PPIB	POLR2A	RPL0	RNA18s5	EEF1A1	YWHAZ
Normfinder	RPL0	GAPDH	RPL22	RNA18s5	EEF1A1	YWHAZ	PPIB	POLR2A
Genorm	EEF1A1 | YWHAZ		RPL0	RNA18s5	RPL22	GAPDH	PPIB	POLR2A
**Recommended comprehensive ranking**	**RPL0**	**RPL22**	**GAPDH**	**EEF1A1**	**YWHAZ**	**RNA18s5**	**PPIB**	**POLR2A**

Comprehensive Ranking

Genes	Geomean of ranking values
RPL0	1.97
RPL22	2.59
GAPDH	2.63
EEF1A1	3.64
YWHAZ	4.12
RNA18s5	4.43
PPIB	5.66
POLR2A	6.73

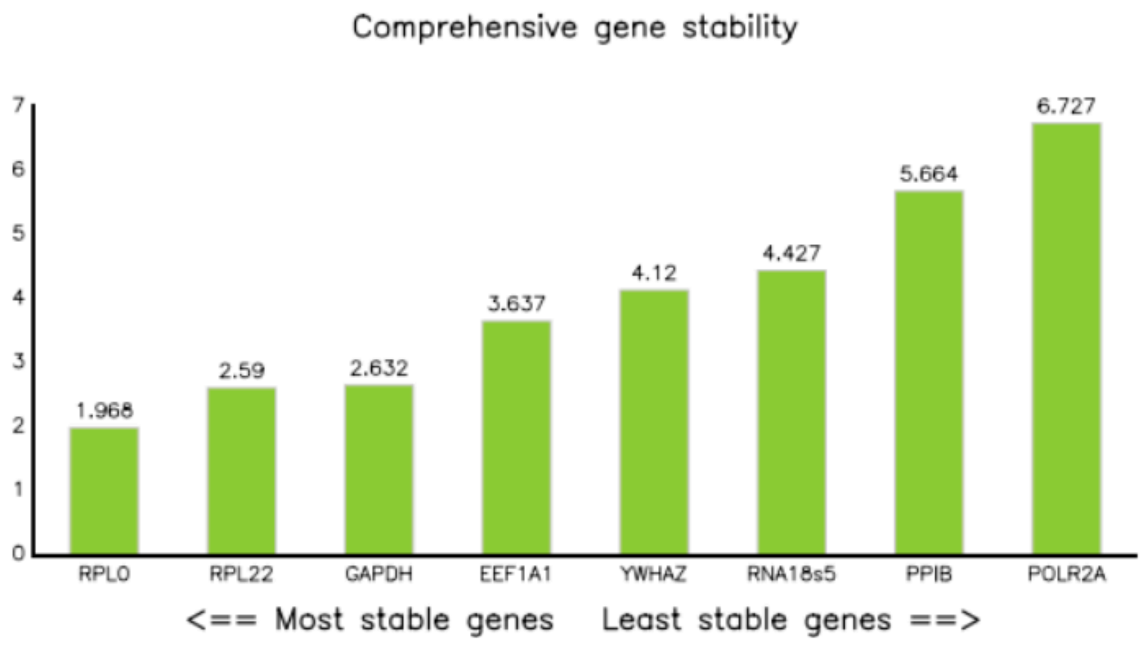

Delta CT

Genes	Average of STDEV
RPL0	0.18
GAPDH	0.20
RPL22	0.20
RNA18s5	0.20
EEF1A1	0.21
YWHAZ	0.24
PPIB	0.26
POLR2A	0.30

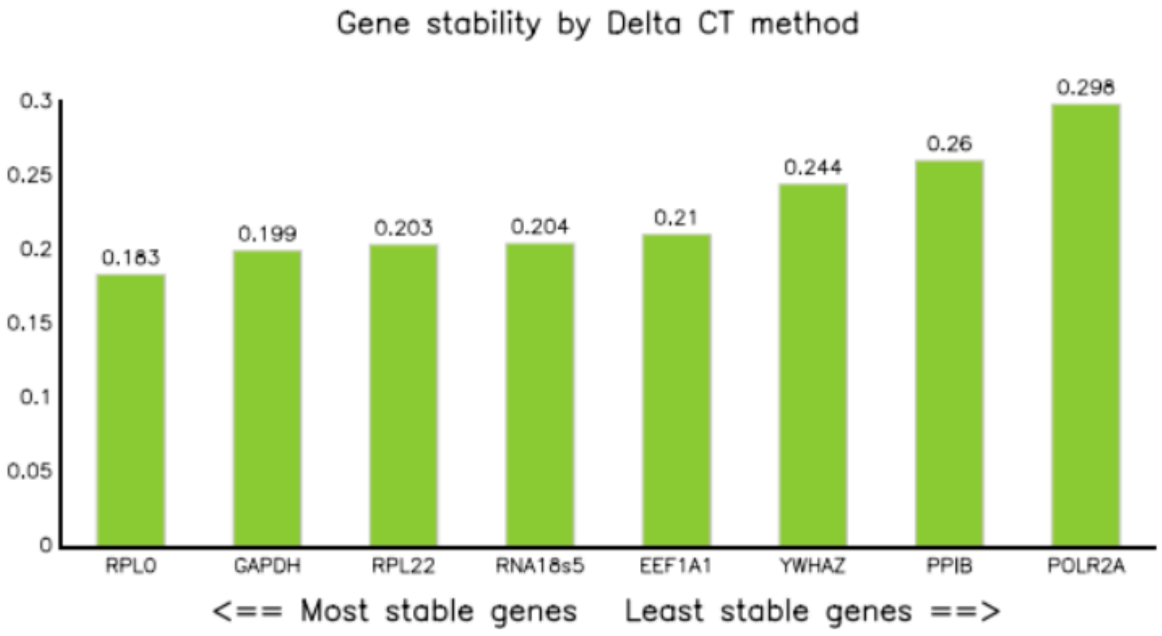

BestKeeper

CP data of housekeeping Genes by BEST KEEPER

	RPL0	EEF1A1	RPL22	PPIB	RNA18s5	YWHAZ	POLR2A	GAPDH
n	8	8	8	8	8	8	8	8
geo Mean [CP]	22.92	19.02	22.79	24.12	9.28	23.13	23.95	20.77
AR Mean [CP]	22.92	19.02	22.79	24.12	9.29	23.14	23.95	20.78
min [CP]	22.74	18.75	22.68	23.96	8.93	22.81	23.79	20.52
max [CP]	23.22	19.49	22.95	24.35	9.64	23.64	24.25	20.97
std dev [+/- CP]	0.16	0.22	0.09	0.11	0.19	0.25	0.15	0.10
CV [% CP]	0.71	1.17	0.38	0.45	2.07	1.08	0.61	0.48
min [x-fold]	-1.14	-1.21	-1.08	-1.11	-1.28	-1.25	-1.12	-1.19
max [x-fold]	1.23	1.38	1.12	1.18	1.28	1.42	1.23	1.15
std dev [+/- x-fold]	1.12	1.17	1.06	1.08	1.14	1.19	1.11	1.07

Pearson correlation coefficient (r) by BEST KEEPER

	RPL0	EEF1A1	RPL22	PPIB	RNA18s5	YWHAZ	POLR2A	GAPDH
EEF1A1	0.961	-	-	-	-	-	-	-
p-value	0.001	-	-	-	-	-	-	-
RPL22	0.777	0.822	-	-	-	-	-	-
p-value	0.023	0.012	-	-	-	-	-	-
PPIB	0.345	0.094	-0.118	-	-	-	-	-
p-value	0.403	0.824	0.782	-	-	-	-	-
RNA18s5	0.749	0.851	0.505	-0.072	-	-	-	-
p-value	0.032	0.007	0.202	0.866	-	-	-	-
YWHAZ	0.855	0.959	0.773	-0.153	0.897	-	-	-
p-value	0.007	0.001	0.025	0.718	0.002	-	-	-
POLR2A	-0.384	-0.229	-0.541	-0.462	0.278	-0.010	-	-
p-value	0.347	0.586	0.166	0.249	0.505	0.982	-	-
GAPDH	0.627	0.556	0.140	0.375	0.651	0.526	0.201	-
p-value	0.096	0.153	0.740	0.360	0.080	0.181	0.633	-

Pearson correlation coefficient (r)

BestKeeper vs.	RPL0	EEF1A1	RPL22	PPIB	RNA18s5	YWHAZ	POLR2A	GAPDH
coeff. of corr. [r]	0.891	0.946	0.631	0.071	0.963	0.946	0.063	0.717
p-value	0.003	0.001	0.093	0.866	0.001	0.001	0.882	0.045

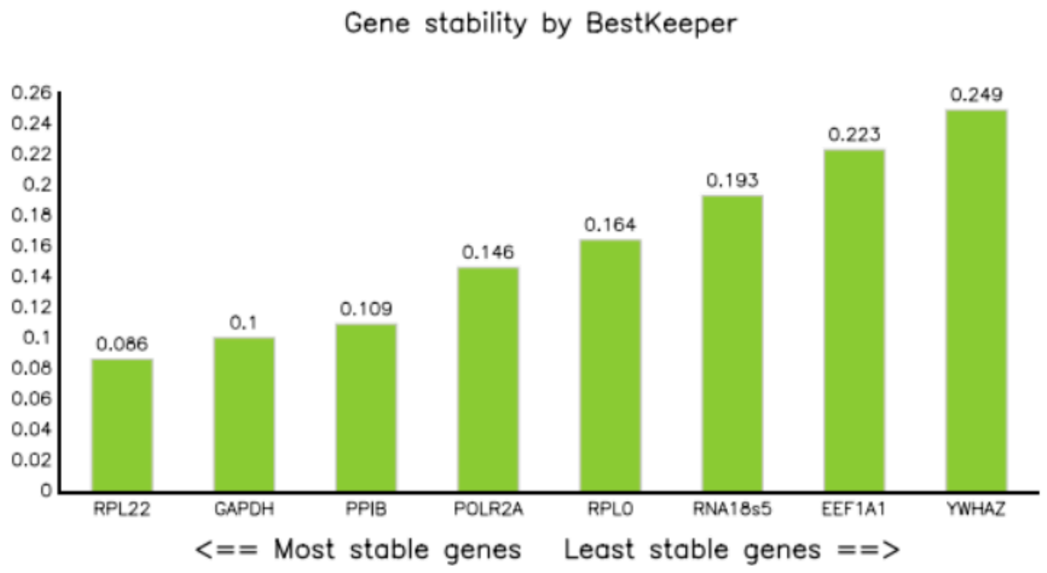

normFinder

Gene name	Stability value
RPL0	0.076
GAPDH	0.102
RPL22	0.111
RNA18s5	0.119
EEF1A1	0.157
YWHAZ	0.208
PPIB	0.217
POLR2A	0.264

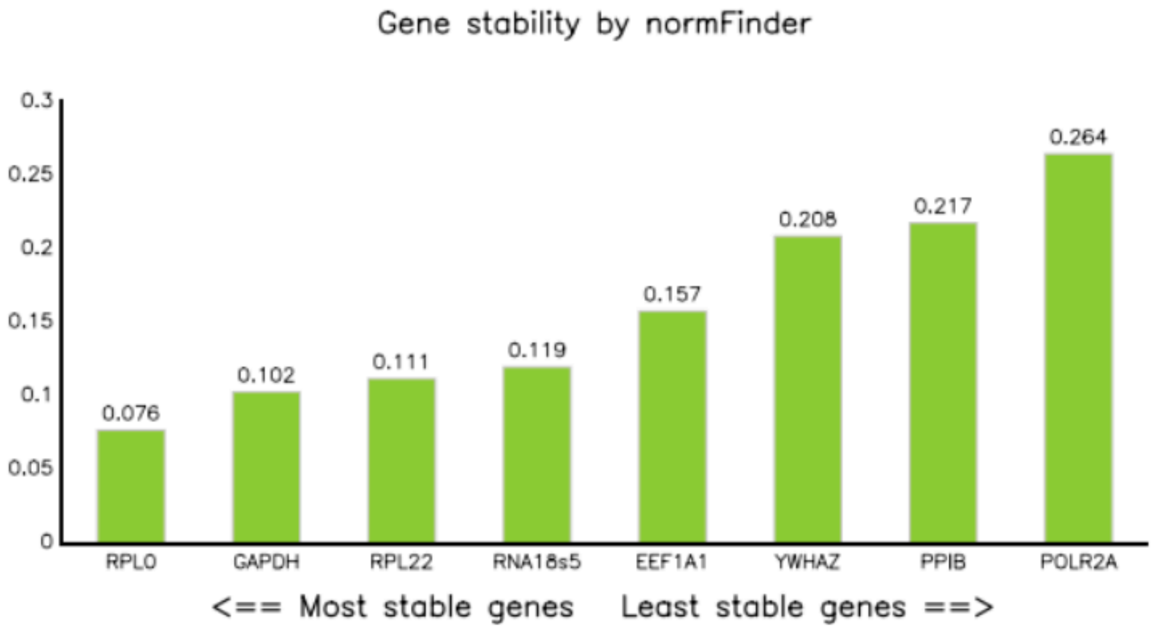

Genorm

Gene name	Stability value
EEF1A1 | YWHAZ	0.096
RPL0	0.128
RNA18s5	0.142
RPL22	0.166
GAPDH	0.179
PPIB	0.201
POLR2A	0.225

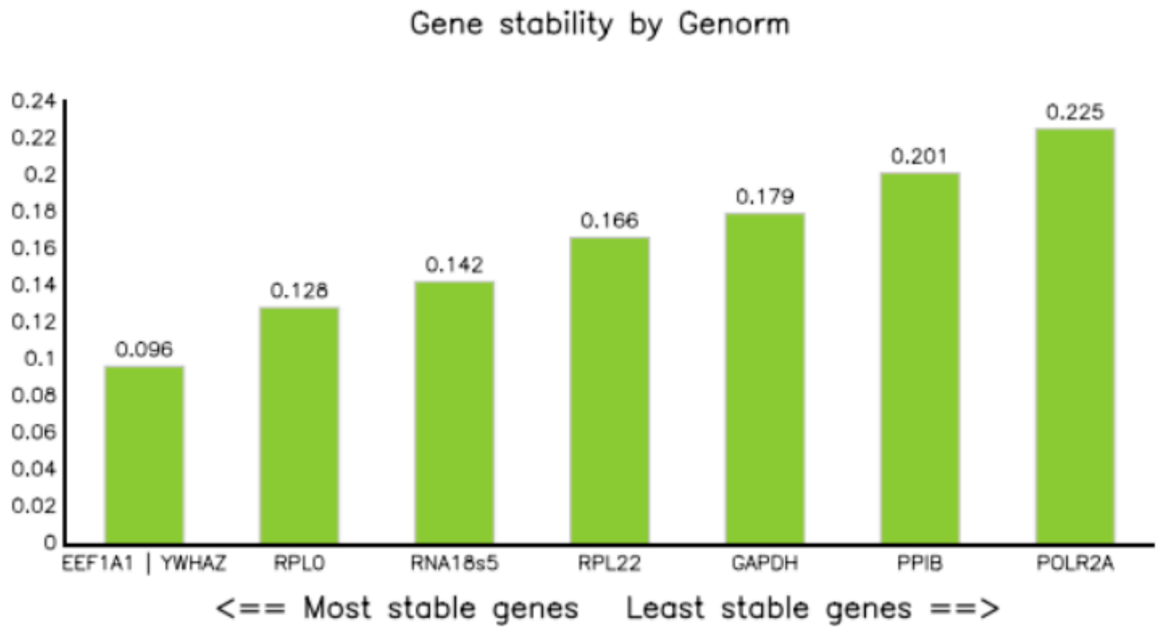

Supplement: Supplementary file 1 [file cells-13-01751-s001.zip › Supplementary File S2.pdf]
