# Supplementary material for: Development of a Custom Fluid Flow Chamber for Investigating the Effects of Shear Stress on Periodontal Ligament Cells
Source: Cells. 2024 Oct 23;13(21):1751. doi: 10.3390/cells13211751 (PMC11545369; doi:10.3390/cells13211751)
Supplement: Supplementary file 1 [file cells-13-01751-s001.zip › Supplementary File S3.pdf]

Supplementary File S3: Western blot data

To the manuscript:

“Investigating the influence of fluid flow shear stress on periodontal ligament cells through a custom-made fluid flow chamber”

R1_Donor 2

Picture of the whole blot

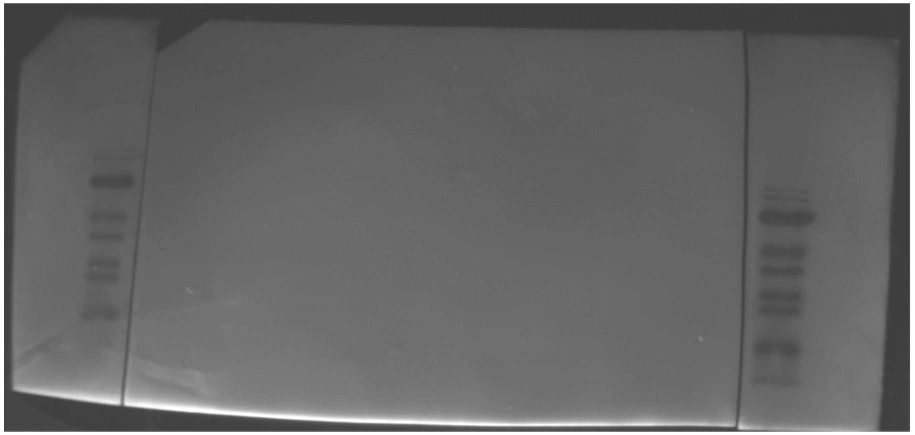

GAPDH

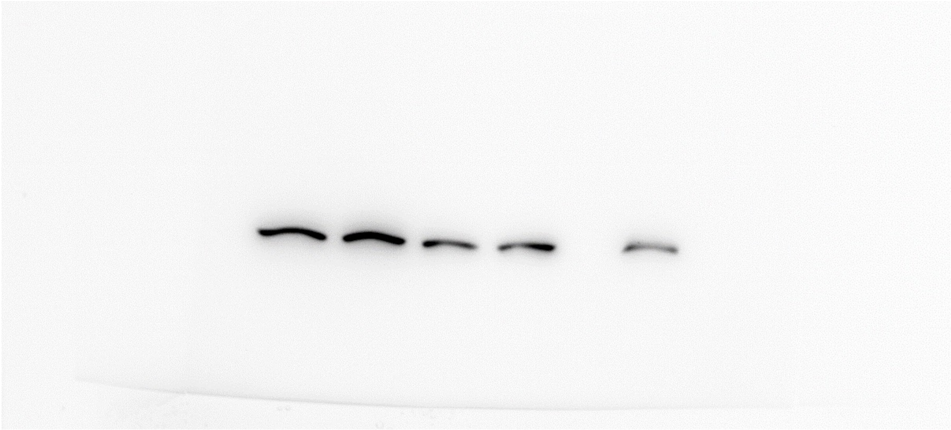

Merged

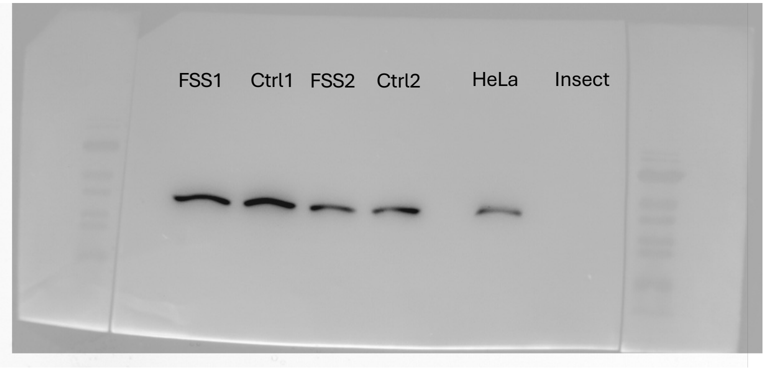

R1_Donor 2

Picture of the whole blot

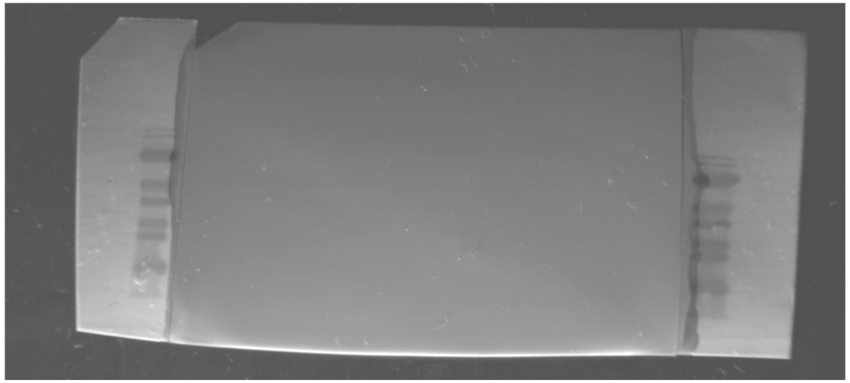

RUNX2

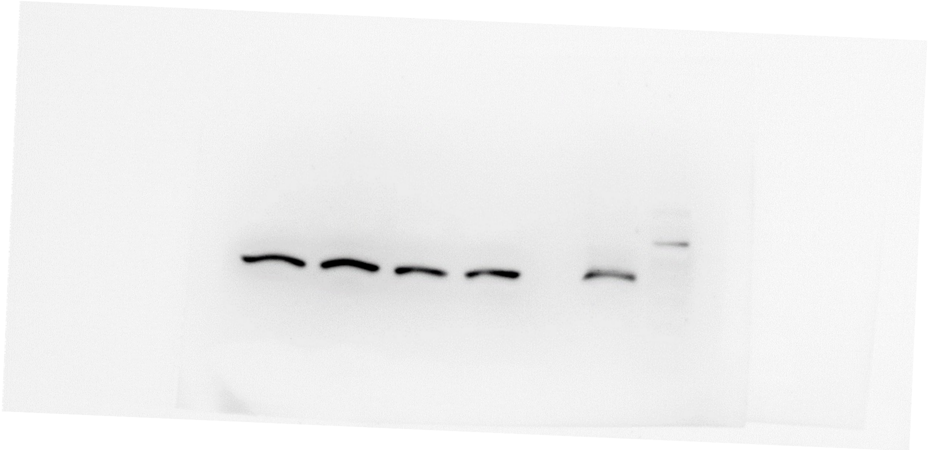

Merged

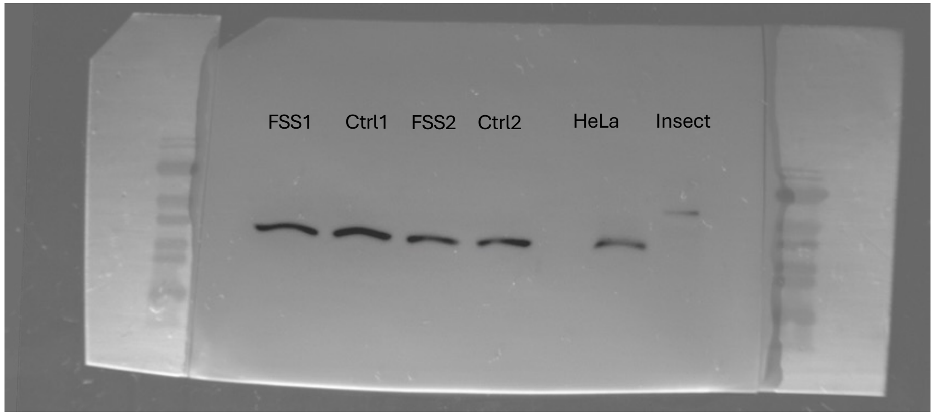

R1_Donor 2

Picture of the whole blot

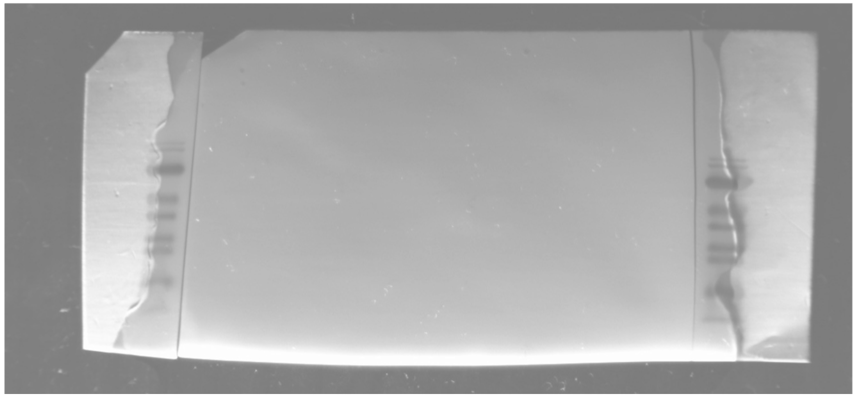

COX 2

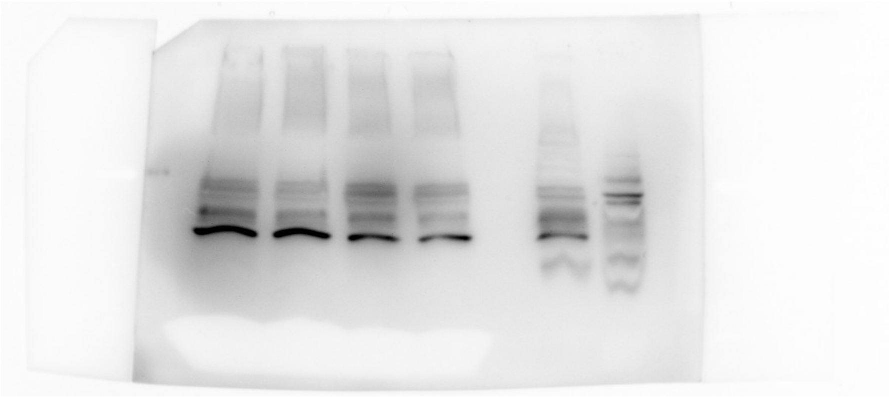

Merged

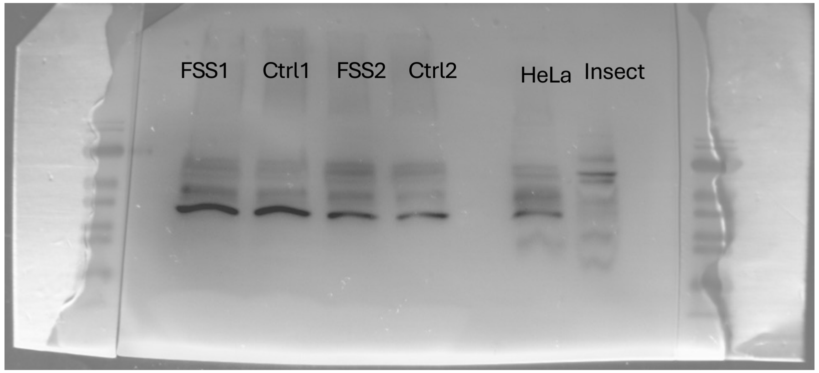

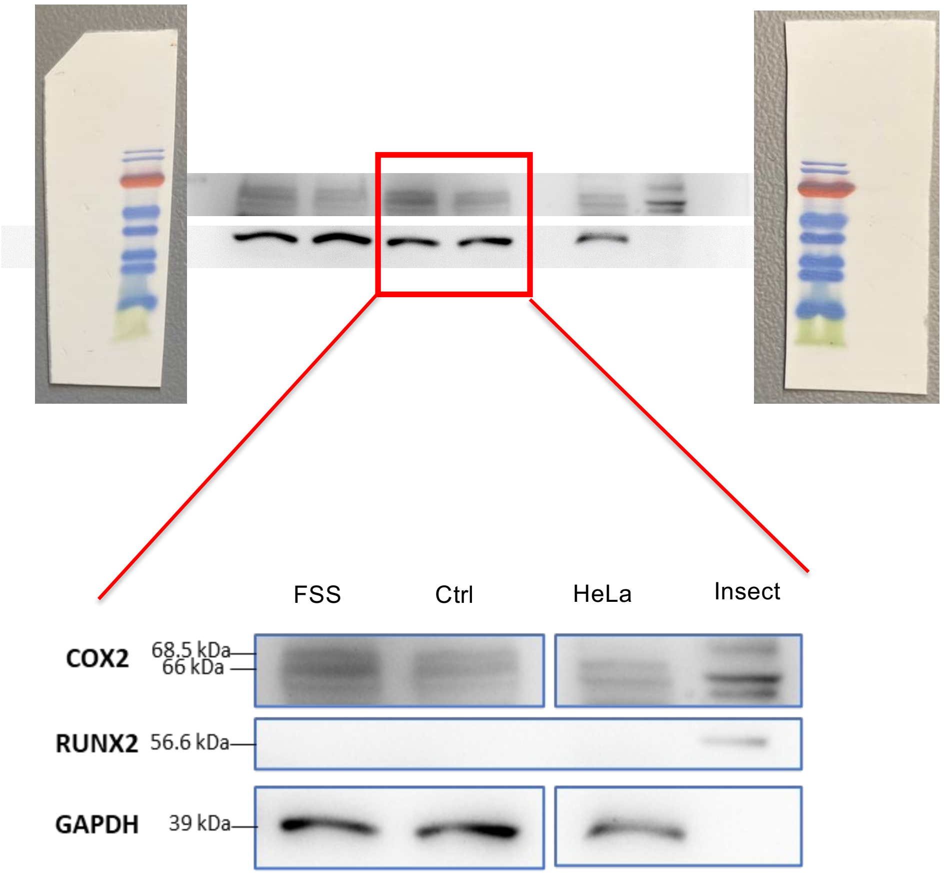
FSS
Ctrl
HeLa
Insect
COX2
68.5 kDa
66 kDa
RUNX2
56.6 kDa
GAPDH
39 kDa

R2_Donor 2

Picture of the whole blot

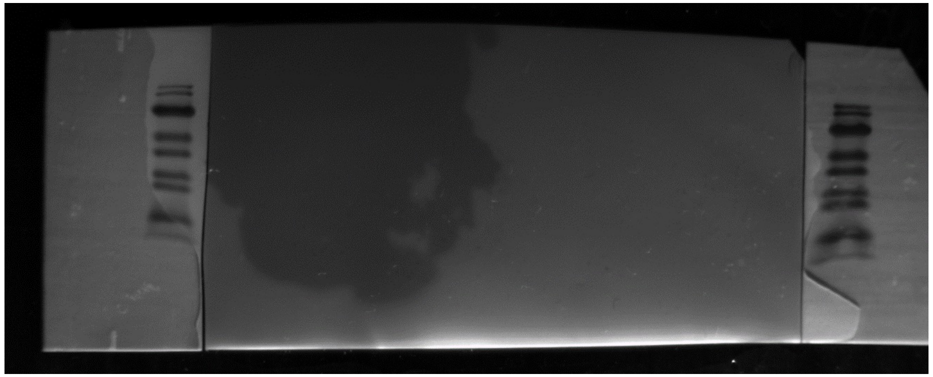

GAPDH

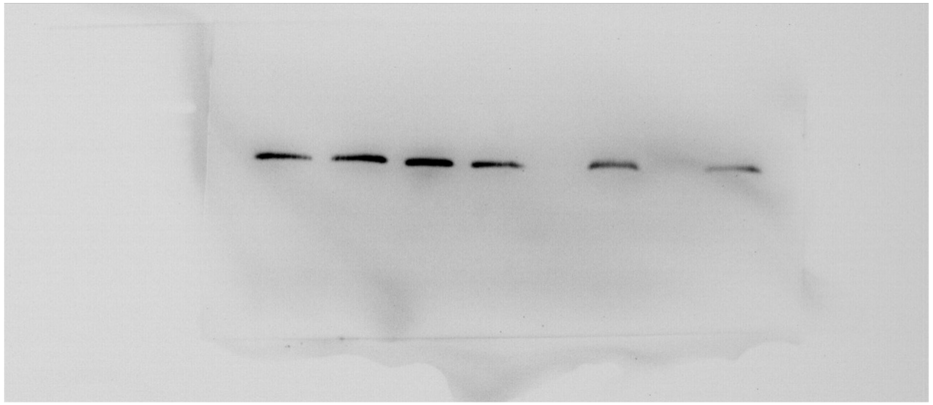

Merged

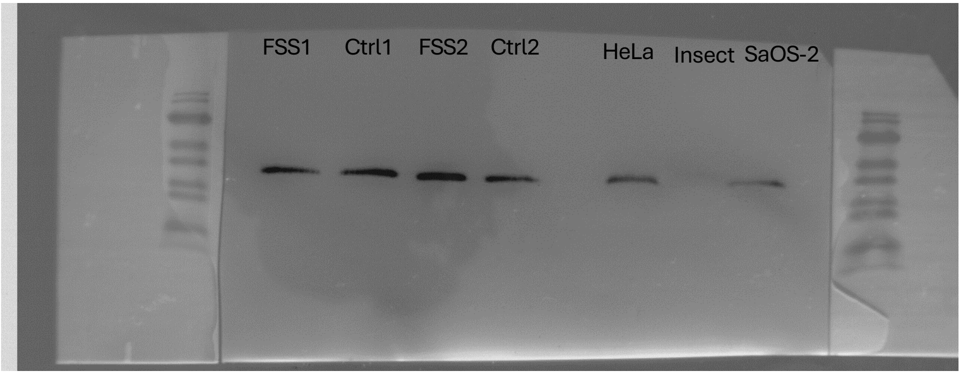

R2_Donor 2

Picture of the whole blot

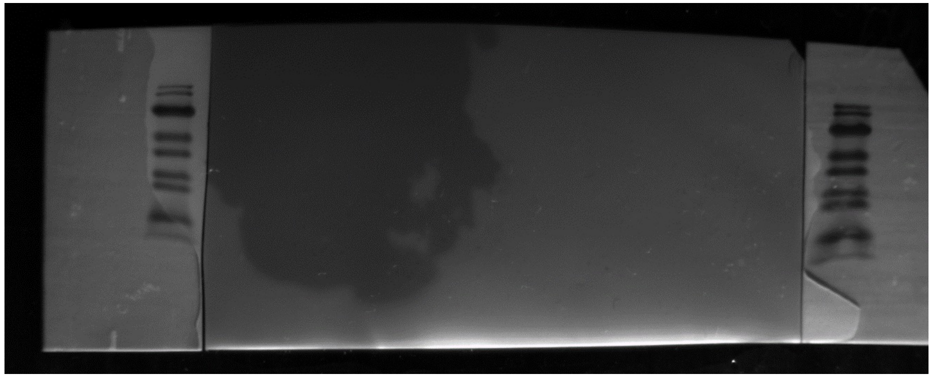

RUNX2

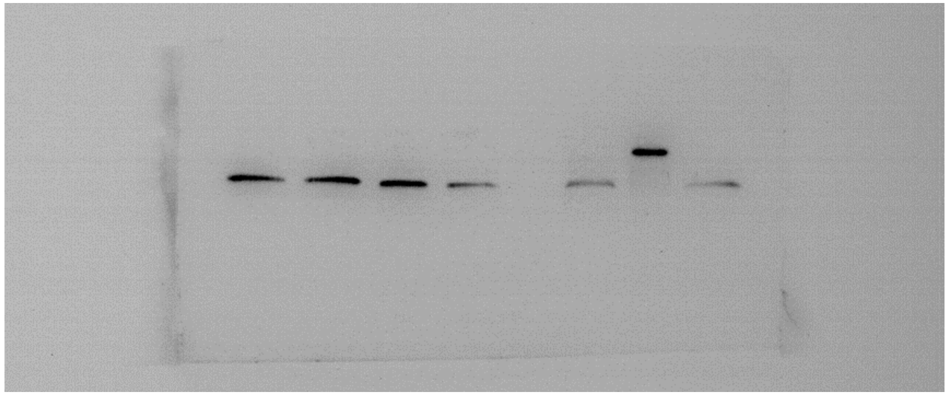

Merged

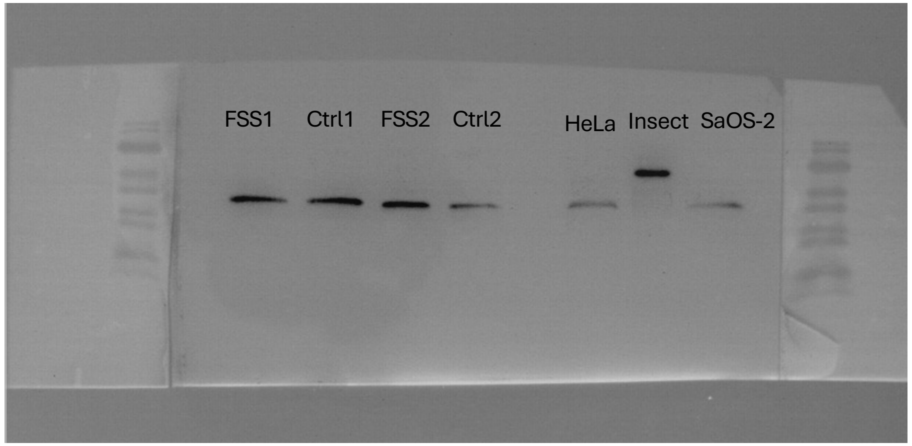

R2_Donor 2

Picture of the whole blot

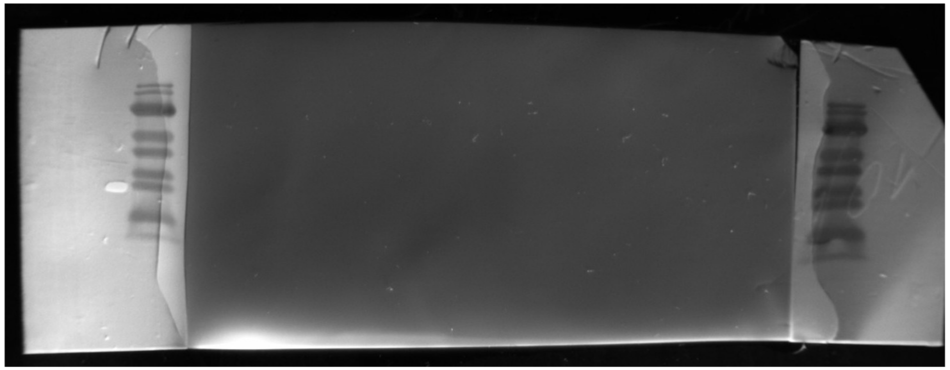

COX2

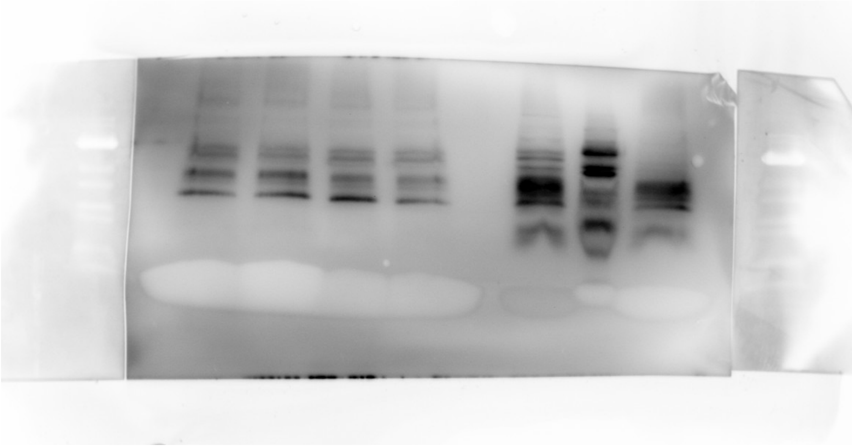

Merged

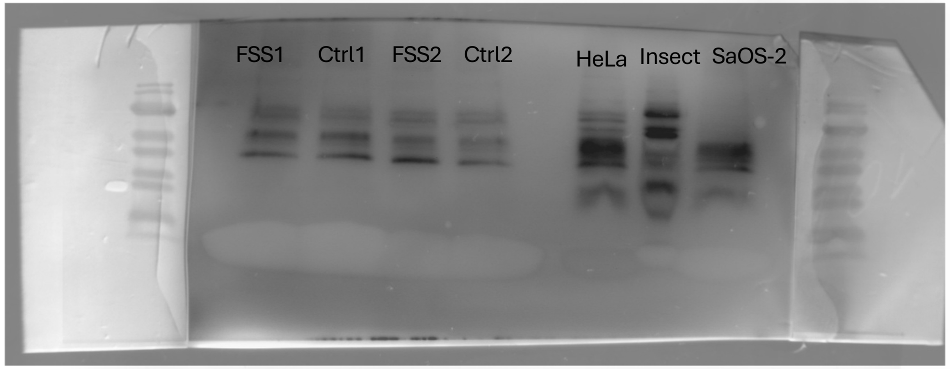

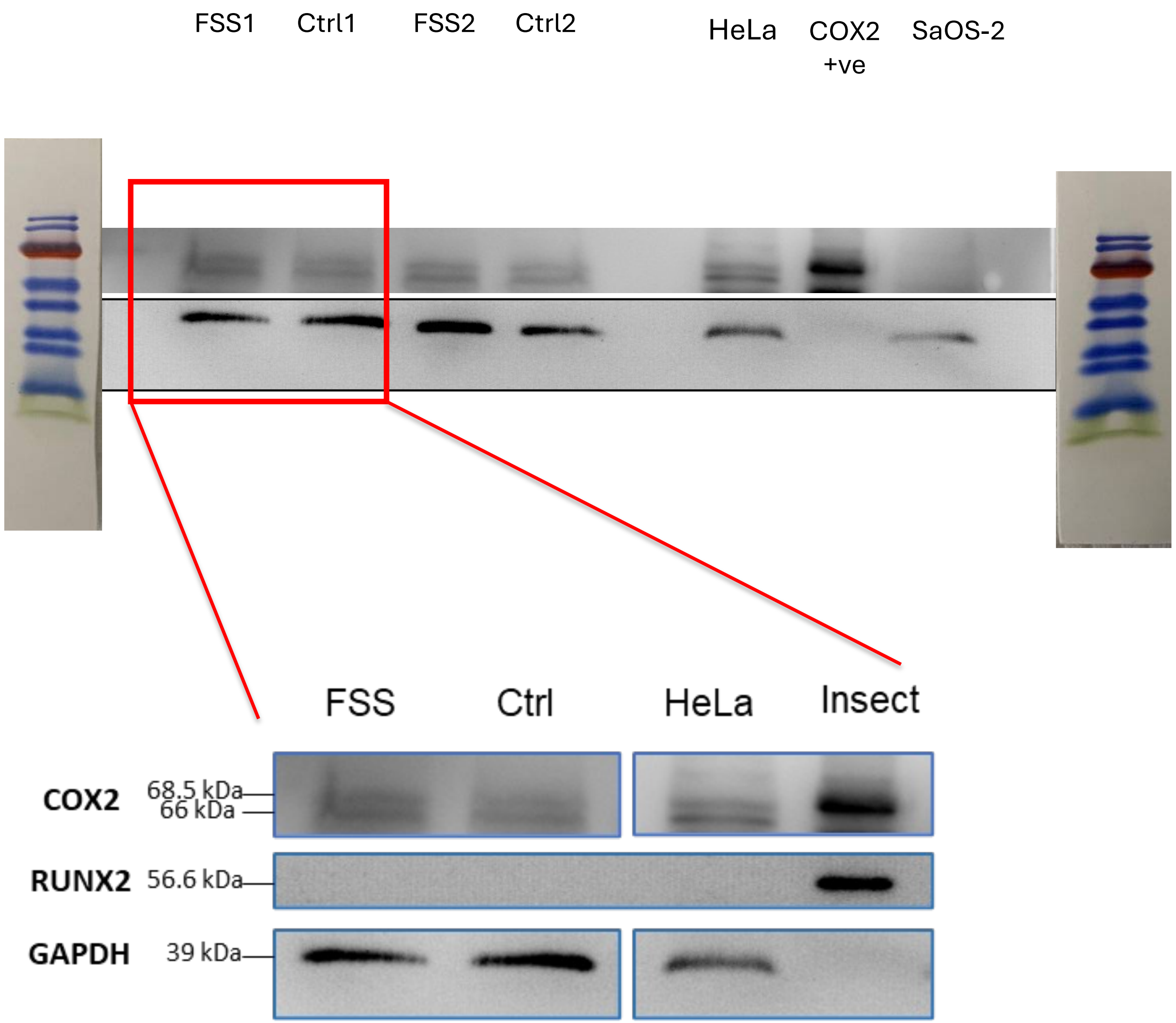
FSS1
Ctrl1
FSS2
Ctrl2
HeLa
COX2 +ve
SaOS-2
FSS
Ctrl
HeLa
Insect
COX2
68.5 kDa
66 kDa
RUNX2
56.6 kDa
GAPDH
39 kDa

R3_Donor 1

Picture of the whole blot

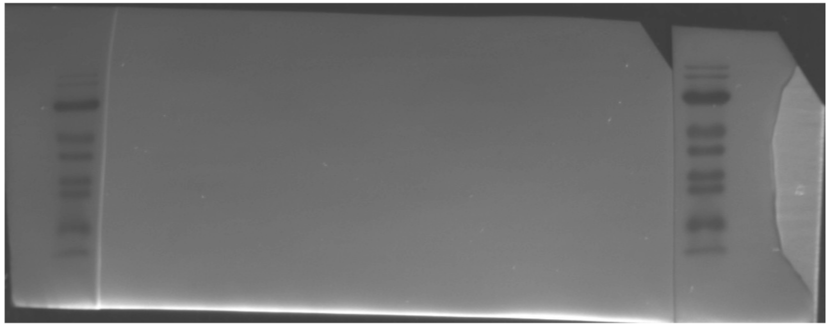

GAPDH

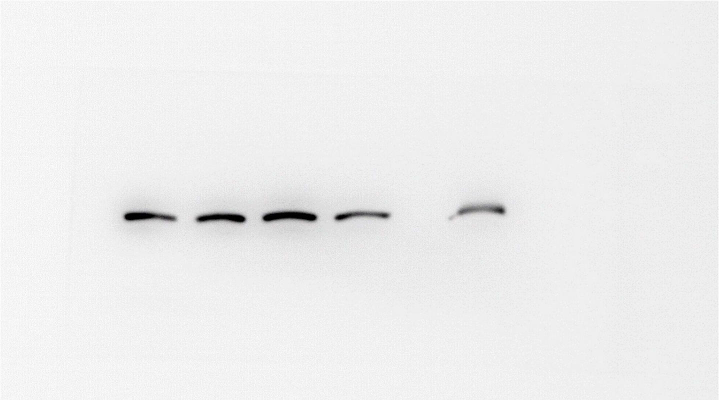

Merged

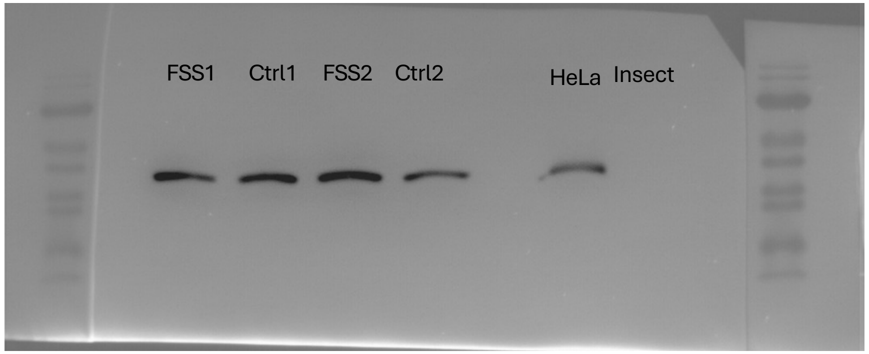

R3_Donor 1

Picture of the whole blot

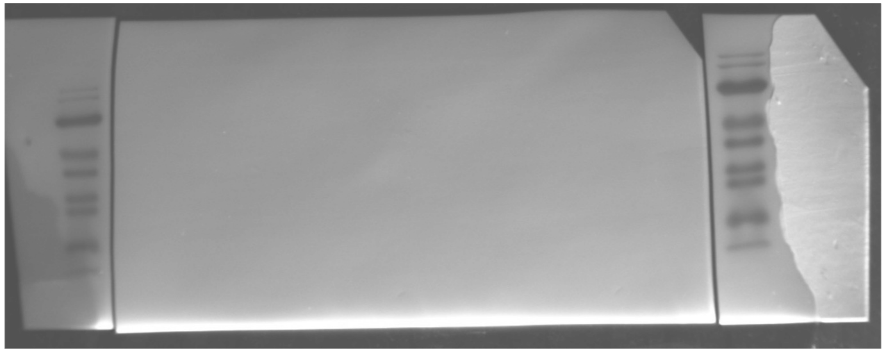

RUNX2

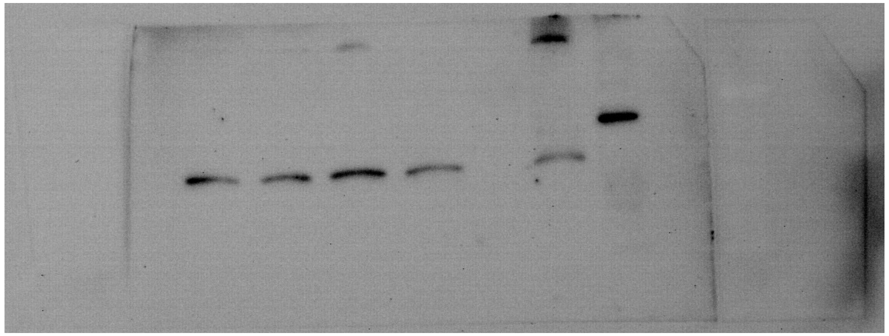

Merged

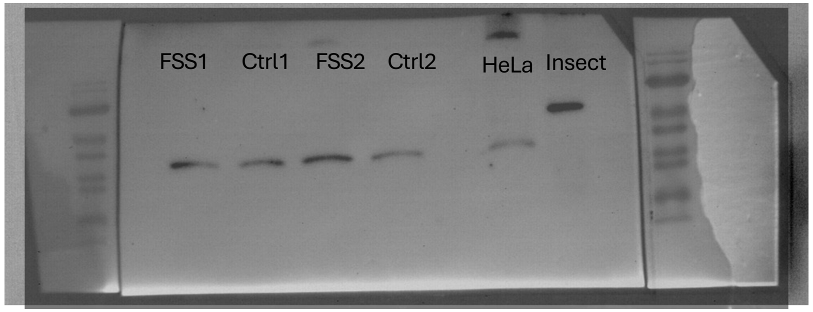

R3_Donor1

Picture of the whole blot

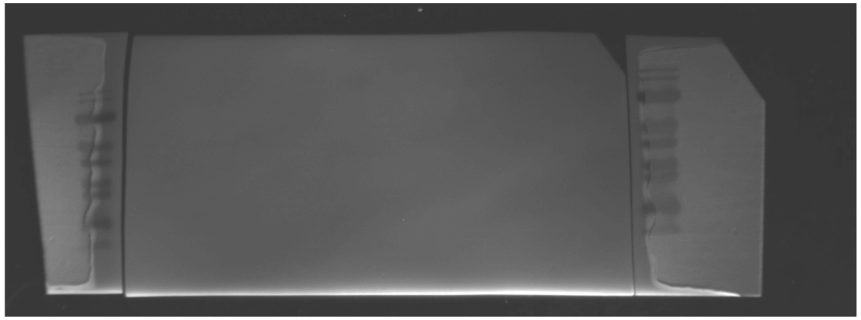

COX2

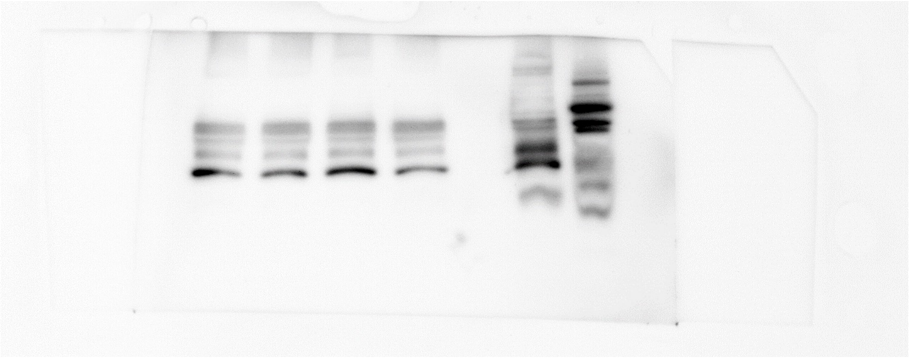

Merged

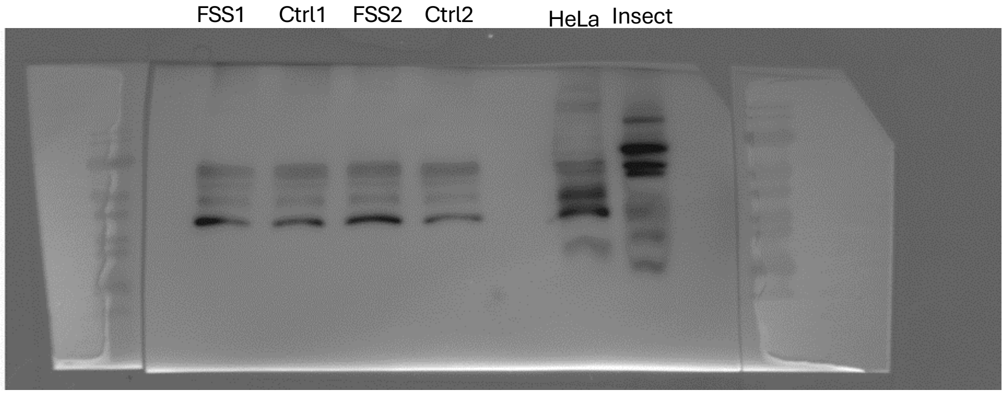

R3_Donor1

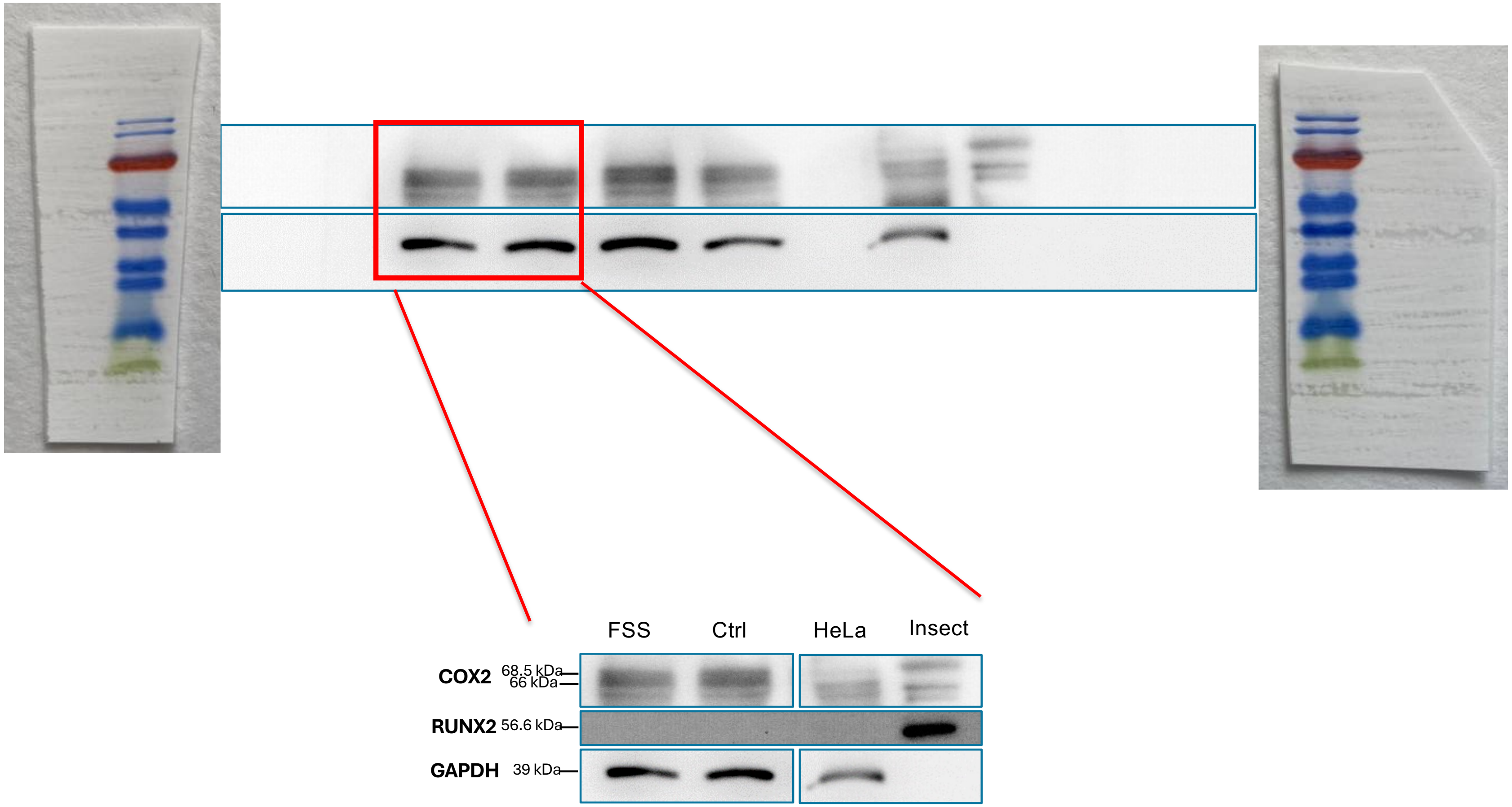
FSS
Ctrl
HeLa
Insect
COX2
68.5 kDa
66 kDa
RUNX2
56.6 kDa
GAPDH
39 kDa

R4_Donor1

Picture of the whole blot

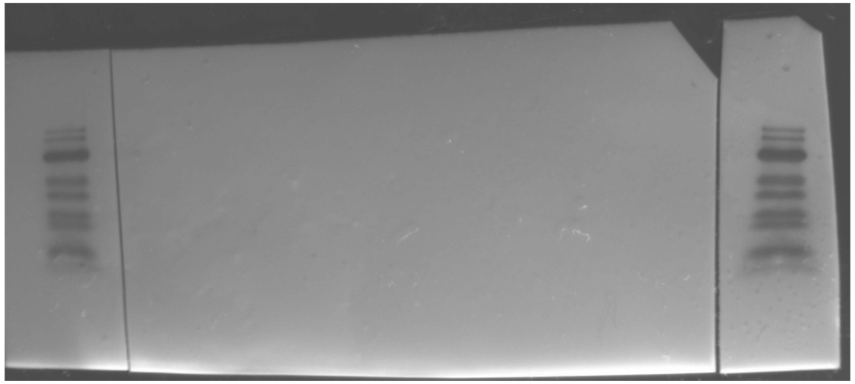

GAPDH

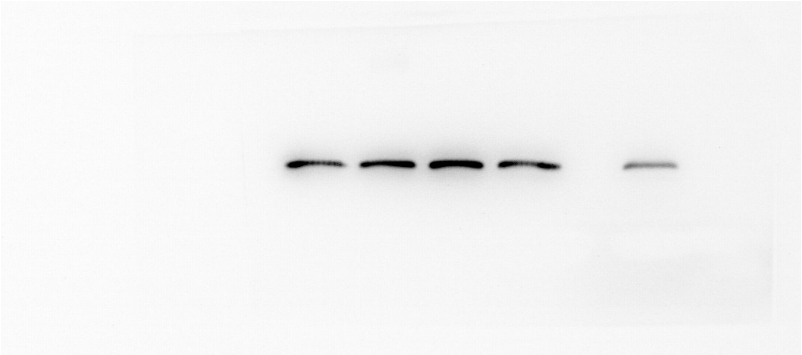

Merged

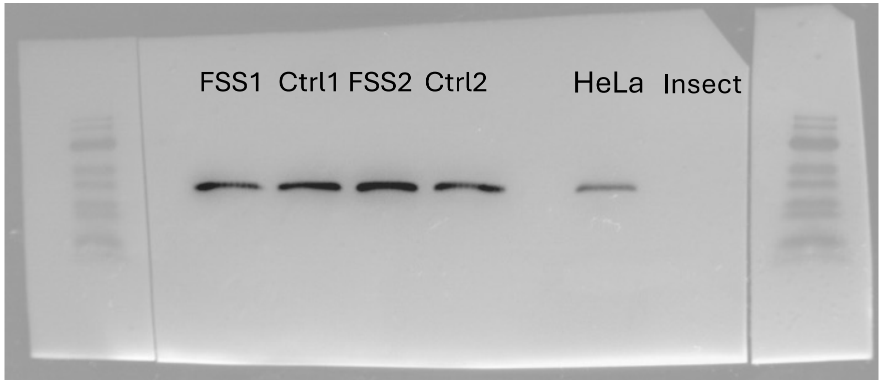

R4_Donor1

Picture of the whole blot

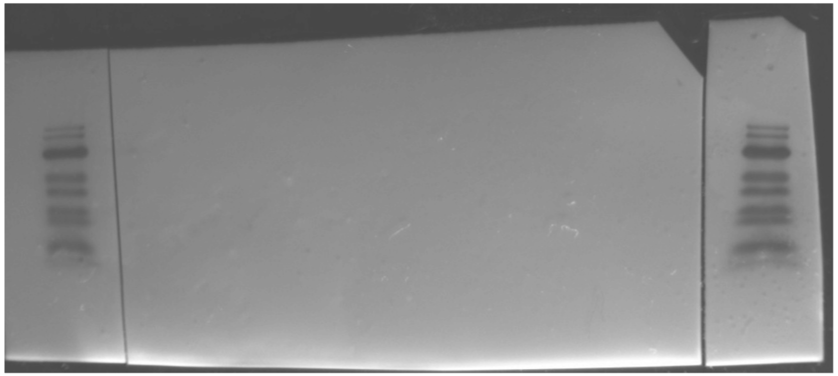

COX2

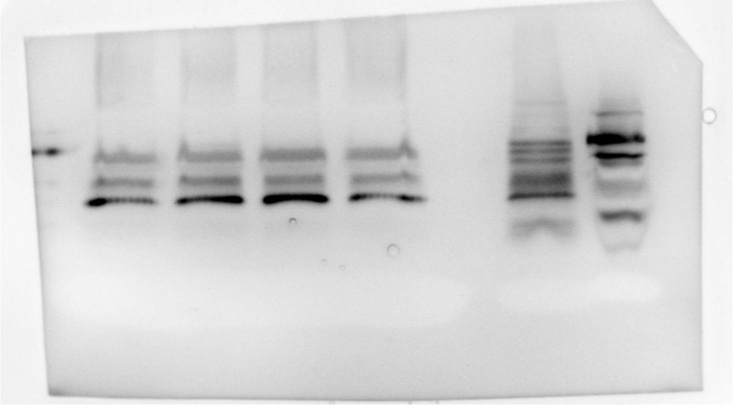

Merged

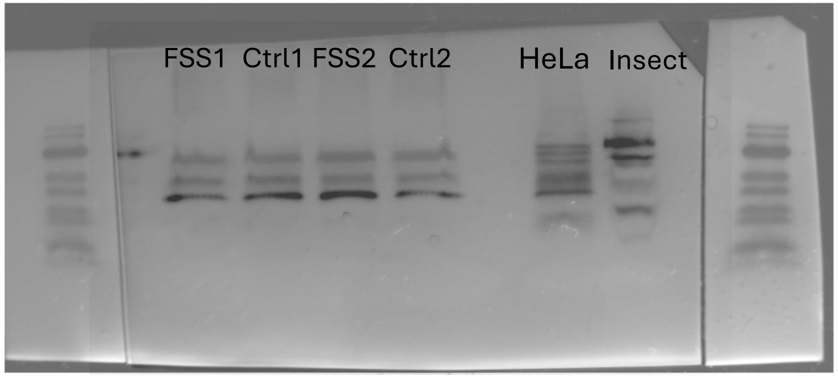

R4_Donor1

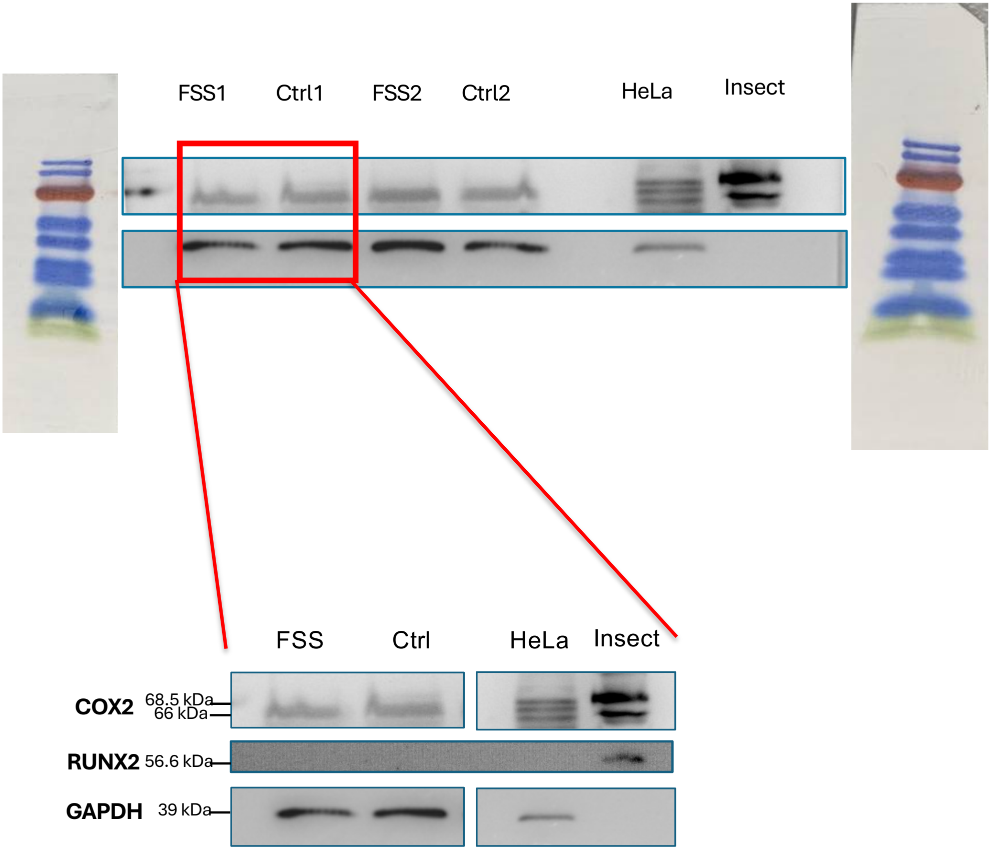
FSS1
Ctrl1
FSS2
Ctrl2
HeLa
Insect
FSS
Ctrl
HeLa
Insect
COX2
68.5 kDa
66 kDa
RUNX2
56.6 kDa
GAPDH
39 kDa

Conclusion

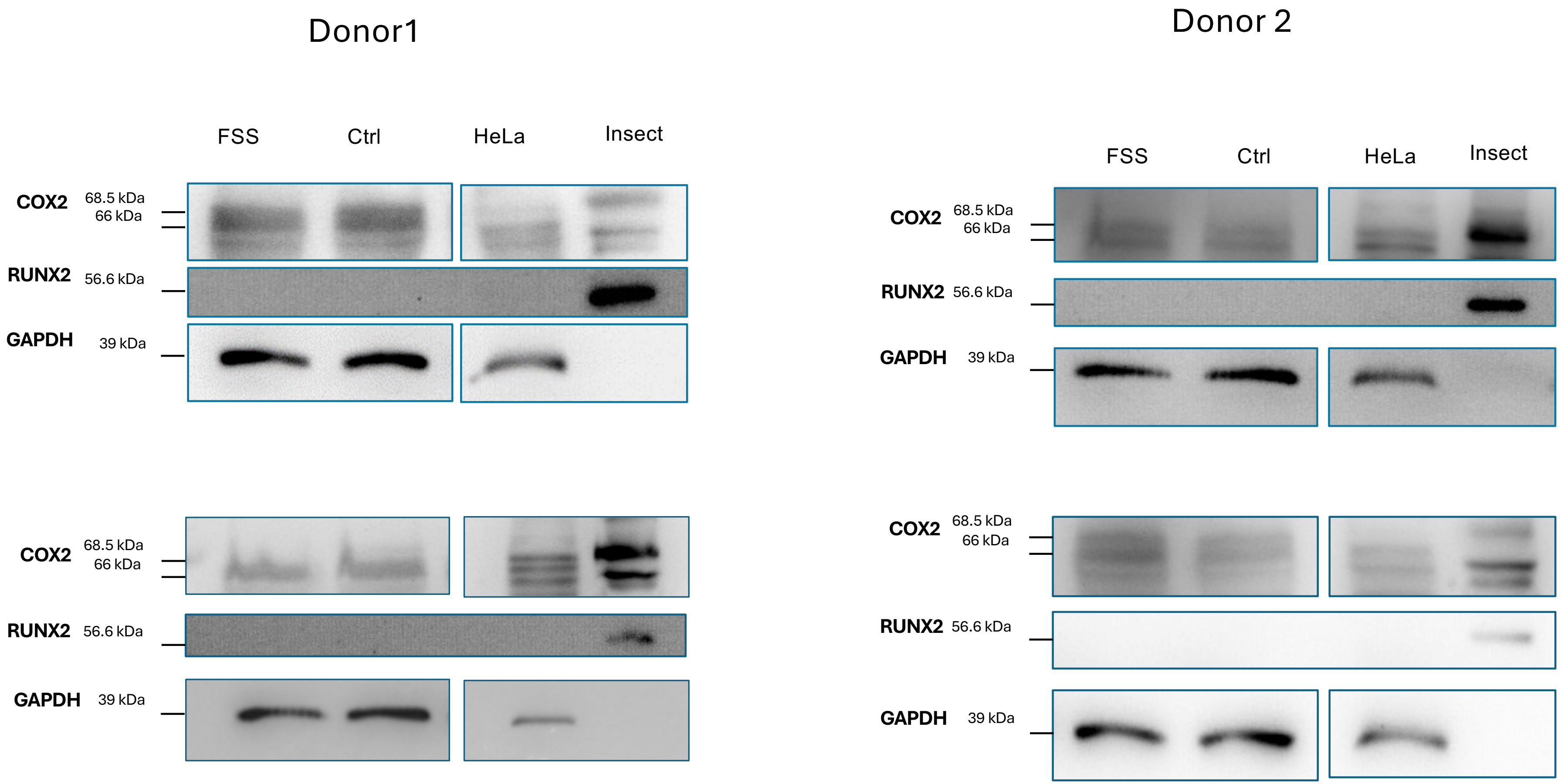

Donor1
FSS
Ctrl
HeLa
Insect
COX2
68.5 kDa
66 kDa
RUNX2
56.6 kDa
GAPDH
39 kDa
COX2
68.5 kDa
66 kDa
RUNX2
56.6 kDa
GAPDH
39 kDa
Donor 2
FSS
Ctrl
HeLa
Insect
COX2
68.5 kDa
66 kDa
RUNX2
56.6 kDa
GAPDH
39 kDa
COX2
68.5 kDa
66 kDa
RUNX2
56.6 kDa
GAPDH
39 kDa

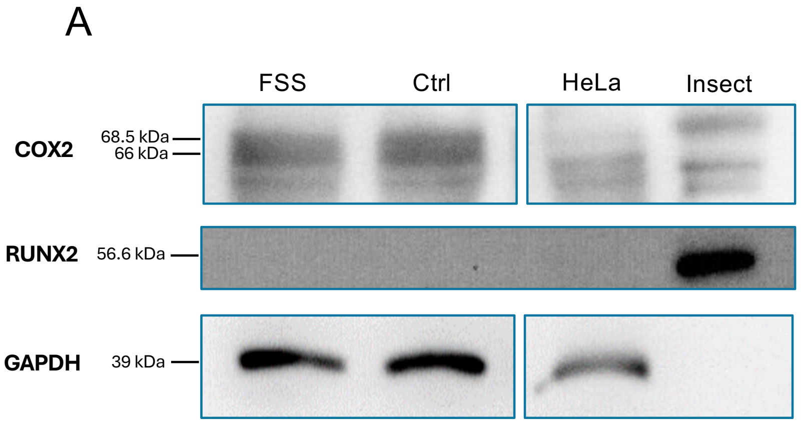
A
FSS
Ctrl
HeLa
Insect
COX2
68.5 kDa
66 kDa
RUNX2
56.6 kDa
GAPDH
39 kDa
B
FSS
Ctrl
HeLa
Insect
COX2
68.5 kDa
66 kDa
RUNX2
56.6 kDa
GAPDH
39 kDa
